# Internet-delivered cognitive behavioral therapy and FODMAP diet for adults with irritable bowel syndrome: A four-arm randomized controlled trial

**DOI:** 10.1016/j.invent.2026.100949

**Published:** 2026-04-26

**Authors:** Camilla Thuen, Robin Maria Francisca Kenter, Jörg Assmus, Elisabeth Kjelsvik Steinsvik, Linn Anja Slåke Vikøren, Gülen Arslan Lied, Birgitte Berentsen

**Affiliations:** a Center for Nutrition, Department of Clinical Medicine, University of Bergen, Bergen, Norway; b Research Centre for digital mental health services, Division of Psychiatry, Haukeland University Hospital, Bergen, Norway; c Centre for Clinical Research, Haukeland University Hospital, Bergen, Norway; d Department of Clinical Medicine, University of Bergen, Bergen, Norway; e Department of Medicine, Section of Gastroenterology, Haukeland University Hospital, Bergen, Norway; f Department of Medicine, Section of Clinical Nutrition, Haukeland University Hospital, Bergen, Norway

**Keywords:** Irritable bowel syndrome, Functional gastrointestinal disorders (FGID), Disorders of gut-brain interaction (DGBI), Digital somatic treatment, Digital treatment program, eHealth

## Abstract

**Background:**

Irritable bowel syndrome (IBS) is a prevalent gastrointestinal disorder associated with reduced quality of life and psychological distress. Although several effective self-management interventions exist, patient access is often limited. Internet-delivered interventions may enhance accessibility and scalability. We aimed to evaluate the effectiveness of a CBT-based module, a FODMAP diet module, or their combination, when added to internet-delivered general patient education, compared to general patient education alone, on IBS symptom severity in adults.

**Methods:**

In this four-arm randomized controlled trial, 642 adults with IBS were allocated to (1) internet-delivered general patient education alone (control) or with the addition of (2) a CBT-based module, (3) a FODMAP diet module, or (4) both the CBT-based module and the FODMAP diet module. All groups could receive personalized guidance from a clinical dietitian via the platform's asynchronous messaging function. The primary outcome was the proportion of IBS symptom responders at 3 months, defined as a ≥ 50-point reduction on the IBS severity scoring system. Secondary outcomes included IBS-related quality of life, psychological symptoms, module adherence, and treatment satisfaction.

**Results:**

Of 557 participants who received their allocated intervention, 373 (67%) completed questionnaires at the primary endpoint at 3 months. Responder rates were comparable across all groups at 3 months ranging from 42.6% to 45.1%, with no significant between-group differences in the primary outcome (OR (95% CI) = 0.97 (0.54 to 1.72), *p* = 0.989). Adherence to the content-specific modules was low, with 9–20% of participants completing the CBT-based and/or FODMAP diet module. All groups showed within-group improvements in IBS symptoms and IBS-related quality of life in secondary continuous analyses, but these did not translate into between-group effects.

**Conclusion:**

All intervention groups, including internet-delivered general patient education alone and general patient education with an additional CBT-based and/or FODMAP diet module, demonstrated clinically and statistically meaningful improvements in IBS symptoms and IBS-related quality of life. Although no additional benefit of the content-specific modules was observed, this finding should be interpreted in the context of the low adherence to these modules. These findings highlight the value of low-intensity internet-delivered interventions for IBS within resource-constrained health systems and emphasize the importance of addressing adherence and engagement in future research.

**Trial registration:**

ClinicalTrials.gov ID: NCT06117865. Registered 31.10.2023.

## Highlights

• Relative effectiveness of different configurations of an internet‑delivered IBS intervention was compared under equivalent delivery conditions.
• Lack of additional benefit from content‑specific modules should be interpreted in the context of low adherence to these modules.
• Findings highlight the value of low‑intensity internet‑delivered interventions for IBS in resource‑constrained health systems.

## 1 Introduction

Irritable bowel syndrome (IBS) is a chronic condition involving recurrent abdominal pain and changes in stool form and/or frequency (Ford et al., 2020). Defined by the Rome III and IV criteria, the global prevalence is estimated to be between 4% and 9%, and the condition is associated with reduced quality of life, increased anxiety and depression and impaired work ability (Oka et al., 2020; Camilleri, 2021; Mearin et al., 2016). Despite the absence of a cure for IBS, several self-management interventions offer proven therapeutic benefits (Camilleri, 2021).

### 1.1 Management for adults with IBS

Current clinical guidelines emphasize general patient education as an essential pillar, with particular focus on reassurance and an individualized approach to management (Lacy et al., 2021; Vasant et al., 2021; Ford et al., 2017). First-line management should include information about general dietary advice, the benefits of regular exercise and stress reduction strategies (Vasant et al., 2021; McKenzie et al., 2016). For some patients, pharmacological treatments targeting diarrhea or constipation may be considered (Lacy et al., 2021).

A diet with reduced intake of fermentable oligosaccharides, disaccharides, monosaccharides, and polyols, namely the FODMAP diet, is recommended as a second-line dietary intervention (Lacy et al., 2021; Vasant et al., 2021; McKenzie et al., 2016). These carbohydrates are poorly absorbed in the small intestine resulting in increased small intestinal water volume and colonic gas production, triggering gastrointestinal symptoms in individuals with visceral hypersensitivity (Vasant et al., 2021). Adherence to all phases of the FODMAP diet is recommended to maintain a diverse diet, but the reintroduction and maintenance phase is often poorly implemented without support from a dietitian (Tuck et al., 2020).

Psychological interventions such as cognitive behavioral therapy (CBT), relaxation training, and hypnotherapy have also demonstrated effect (Black et al., 2020; Laird et al., 2016). Different CBT protocols for IBS patients exist, often sharing core principles that address how thoughts, emotions and behaviors are bidirectionally related to IBS symptoms (Laird et al., 2017).

Although effective interventions exist for IBS, the condition is still commonly regarded by general practitioners as difficult to diagnose and manage (Heidelbaugh et al., 2025). This is reflected in high healthcare costs associated with IBS, with estimates of €2889 per patient annually and national healthcare burdens reaching billions (Tornkvist et al., 2021; Flacco et al., 2019). Despite extensive healthcare use, many patients with IBS report limited satisfaction with the care they receive and describe a prolonged search for effective symptom relief, often marked by repeated disappointment (El-Salhy et al., 2025; Harvey et al., 2018). Combined with limited patient access to integrated care, these challenges highlight the need for more accessible and scalable low-threshold interventions (Chey et al., 2021).

### 1.2 Internet-delivered management for adults with IBS

Internet-delivered evidence-based interventions offer scalability, reduced waiting times, and geographic flexibility, while potentially reducing healthcare system burden. Evidence support that internet-delivered interventions improve gastrointestinal symptoms, psychological indices and quality of life in patients with IBS, with comparable effectiveness to face-to-face delivery (Laird et al., 2016; D'Silva et al., 2025). While previous studies have demonstrated treatment effects of internet-delivered CBT (Wallen et al., 2025; Ljotsson et al., 2011; Pathipati et al., 2024), FODMAP diet (Carbone et al., 2022; Dimidi et al., 2023) and a self-management program (Tayama et al., 2024), no trials have compared different internet-delivered interventions within the same cohort and under equivalent delivery conditions. This leaves a gap in understanding the relative effectiveness of different configurations of internet-delivered interventions when delivered under equivalent delivery conditions.

## Objectives

The primary objective of this study was to evaluate the effectiveness of a CBT-based module, a FODMAP diet module, or their combination, when added to internet-delivered general patient education, compared to general patient education alone, on IBS symptom severity in adults. Secondary objectives included assessing effectiveness on IBS-related quality of life, psychological indices, as well as treatment satisfaction and adherence.

## 2 Methods

### 2.1 Ethics

This study is reported in accordance with the CONSORT statement for randomized trials, and a completed checklist is available as Supplementary File 1 (Hopewell et al., 2025). Ethics approval was received from the Regional Committees for Medical and Health Research Ethics Western Norway (ID # 630038) and the participants provided their written informed consent prior to participation. The study protocol was registered at ClinicalTrials.gov (ID #NCT06117865). Following registration, one amendment was made. The Working Alliance Inventory was not administered, based on participant feedback indicating that the questionnaire was difficult to understand.

Patient representatives were involved in multiple stages of the study, and patient feedback informed the development of the digital treatment program, the study design, participant information materials, and recruitment strategies. They also provided input to the interpretation of study findings.

### 2.2 Study setting

This study was conducted within an established internet-delivered treatment program for IBS at Haukeland University Hospital, Bergen, Norway. The program consists of five modules with a multidisciplinary approach and was developed by the National Competence Center for Functional Gastrointestinal Diseases and has been available to patients since 2016. The internet-delivered treatment program has previously been evaluated in comparison with the onsite multidisciplinary group-based education program as the alternative treatment option (Berentsen et al., 2025). Additionally, the factors influencing the implementation process of the program have been evaluated (Thuen et al., 2025).

### 2.3 Study design

This study was a four-armed randomized controlled trial (RCT) with an active control and three intervention groups, with participants randomly allocated in a 1:1:1:1 ratio to one of four groups. The randomization sequence was generated in GraphPad (GraphPad Software, San Diego, CA, USA) using block randomization with a block size of 40 by author CT.

#### 2.3.1 Patient recruitment and screening

Patient recruitment took place between December 2023 and March 2025 through multiple channels, including social media, national newspapers and magazines, local radio interviews, articles in journals for gastroenterologists and general practitioners, and a feature in the national IBS patient organization's journal. Interested individuals were directed to an information page with a screening form consisting of the eligibility criteria. The screening form collected data using REDCap electronic data capture tools hosted at Haukeland University Hospital, Bergen, Norway (Harris et al., 2009; Harris et al., 2019). Eligibility criteria are described in Table 1.

**Table 1:** Eligibility criteria.

Category	Criterion
Diagnosis	Diagnosed with IBS in primary or secondary healthcare.
Age	18–70 years.
Diagnostic criteria	Meets Rome IV criteria for IBS.
Medical history	No known history or current presence of: – Symptomatic endometriosis – Celiac disease – Type 1 or 2 diabetes mellitus – Malignant disease (excluding basal cell carcinoma) – Severe psychiatric disorder (e.g., treatment-requiring schizophrenia or bipolar disorder with functional impairment) – Alcohol or drug abuse – Inflammatory bowel disease (including microscopic colitis) – Diverticulitis or ileus – Major abdominal surgery (except appendectomy, cholecystectomy, cesarean section, and hysterectomy) Negative colonoscopy within the last 5 years (if >55 years) Not pregnant and not planning pregnancy within the next 6 months No unexplained symptoms such as: – Night sweats (repeated episodes soaking nightclothes or bedding) – Unintentional weight loss ≥4.5 kg or ≥ 5% of normal body weight within <6 months – Blood in stool
Technological access	Access to a national electronic ID system (BankID) and a tablet, smartphone, or personal computer with internet.
Protocol compliance	Able to comply with study protocol requirement and able to read and understand Norwegian.

Note: IBS = Irritable bowel syndrome.

After submission of the screening form, eligible participants were contacted by a phone call by trained personnel (research nurse or dietitian) to confirm and clarify the details provided in the screening form. The information relied on the participants' self-report. However, if uncertainty regarding any eligibility criterion was identified during the screening phone call, participants were again asked to contact their general practitioner to clarify the relevant information prior to inclusion. As part of the study procedures, enrollment and allocation were carried out by the same personnel, who had access to the allocation sequence, during this phone call. Given the characteristics of the internet-delivered interventions and the general patient education, participant blinding was not feasible.

#### 2.3.2 Description of study groups

**Fig. 1:**
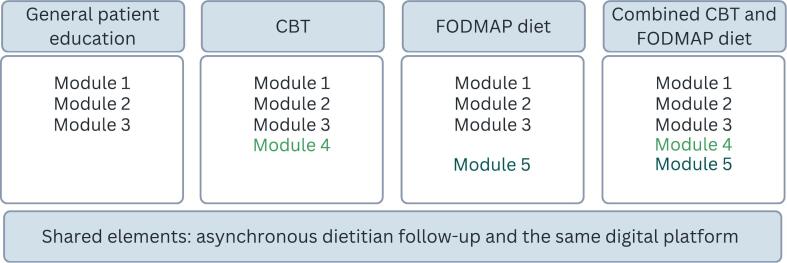
Description of study groups. Note: Module 1: Basic information about IBS (23 pages); Module 2: Information about pain physiology (13 pages); Module 3: General diet and lifestyle advice (14 pages); Module 4: Cognitive behavioral therapy (14 pages of psychoeducational content, 15 pages of exercises, and 3 audio files); Module 5: the FODMAP diet (45 pages, recipe booklet, suggested daily menus, and a structured reintroduction worksheet). CBT = Cognitive behavioral therapy; FODMAP = Fermentable Oligo-, Di-, Mono-saccharides And Polyols.

Participants were allocated to one of four study groups, and the assigned modules corresponded to their treatment allocation (Fig. 1).

After allocation, all participants received access to the electronic platform accessible on smartphone, tablet, or computer (EG CheckWare, Trondheim, Norway). Upon first login, participants were presented with the questionnaires, which had to be completed within 14 days before proceeding to the intervention content. While all intervention modules were technically available in the system from start, access required active navigation past the baseline assessments. Modules were primarily text-based, with some modules offering additional videos and practical exercises (Table 2).

**Table 2:** Description of the modules.

Module	Module objectives	Key topics
Module 1 (Gastroenterology)	Provide a rationale for IBS symptoms, offer reassurance about their condition and a sense of normalization.	What IBS is, how it is diagnosed, how the gastrointestinal system works, and how it can be disrupted in IBS.
Module 2 (Physiotherapy)	Provide an understanding of how pain in IBS develops, why it persists, and normalize the link between IBS and extraintestinal symptoms.	Information about pain physiology, normal physical maladjustments, videos demonstrating how posture and breathing can be affected and how to normalize it.
Module 3 (General lifestyle)	Provide a rationale behind the general diet and lifestyle advice and teach practical skills to implement these.	Guidance on general dietary strategies, with an emphasis on applying these before progressing to more restrictive diets.
Module 4 (CBT)	Provide an understanding of the relationship between psychological factors and IBS and teach practical behavioral techniques to reduce their impact.	Psychological factors in IBS, cognitive diffusion and reperceiving, and practical exercises for symptom observation and reinforcing activities.
Module 5 (FODMAP diet)	Provide a rationale behind the three phases of the FODMAP diet and teach practical skills to implement the diet properly.	Information about how FODMAPs are related to IBS symptoms, the type of foods in which FODMAPs are typically found, detailed guidance on how to restrict foods high on FODMAPs during the elimination phase, emphasis on the importance of the reintroduction phase for establishing a long-term maintenance diet.

Note: CBT = Cognitive behavioral therapy; FODMAP = fermentable oligosaccharides, disaccharides, monosaccharides, and polyols; IBS = Irritable bowel syndrome.

Participants were encouraged to complete the modules within 3 months, although platform access remained available for 6 months. Additionally, all participants could receive personalized asynchronous guidance from a clinical dietitian via the platform's messaging function. Participants were encouraged to use this feature, but guidance was not structured and relied on participant-initiated contact. Automated reminder text messages were sent during the intervention period to prompt questionnaire completion and encourage use of the messaging feature.

##### 2.3.2.1 Internet-delivered general patient education

To account for the anticipated placebo response rate and potential beneficial effects of common therapeutic elements such as therapeutic support, symptom explanation, expectation of improvement, and enhanced self-efficacy, the active control group received access to the general patient education modules (Bosman et al., 2021; Boyce et al., 2003; Cuijpers et al., 2019). These three modules provided basic information about IBS symptoms (23 pages), pain physiology (13 pages), and general diet and lifestyle advice (14 pages). They also included practical tips for implementing these first-line self-management strategies.

##### 2.3.2.2 Internet-delivered CBT

In addition to the first three general patient education modules, the participants in the CBT group received a CBT-based module (14 pages of psychoeducational content, 15 pages of exercises, and 3 audio files). The module was primarily grounded in exposure-based CBT for IBS, with a focus on behavioral strategies and symptom exposure. The CBT-based module was uniquely developed for this program by a clinical psychologist, based on existing evidence on psychological treatment of IBS, demonstrating that symptom control can be achieved through increased acceptance of and exposure to symptoms (Ljotsson et al., 2011; Ljotsson et al., 2014; Axelsson et al., 2023).

##### 2.3.2.3 Internet-delivered FODMAP diet

Participants in the FODMAP diet group received access to a module on the FODMAP diet (45 pages) in addition to the first three modules, covering information about FODMAPs and guidance in how to implement all phases of the diet. Additional menus, recipes and a diary for the reintroduction phase were also provided. The FODMAP module was uniquely created for this program by clinical dietitians, based on existing evidence and recommendations for implementing the FODMAP diet (Whelan et al., 2018).

##### 2.3.2.4 Internet-delivered combined treatment

The CBT-based module and the FODMAP diet are conceptually opposite approaches to managing IBS symptoms (Biesiekierski et al., 2022). While CBT focuses on exposure to symptoms and reducing the impact by shifting attention away from them, the FODMAP diet encourages participants to monitor their symptoms and dietary intake to identify and avoid foods that trigger symptoms. Although each treatment has demonstrated efficacy, there is limited evidence on how these treatments work together or whether they can be integrated into a holistic management of IBS symptoms. To explore the clinical effectiveness of a combined configuration, we included a group that got access to both the CBT-based module and the FODMAP module, in addition to the general patient education.

### 2.4 Measures

All outcome measures were self-reported and collected via questionnaires completed by participants through the same electronic platform used for the digital treatment program (EG CheckWare, Trondheim, Norway), except for module adherence, which was based on platform-generated usage data. Assessment was performed at three time points: baseline, 3 months and 6 months.

#### 2.4.1 Primary outcome measure

##### 2.4.1.1 Irritable bowel syndrome severity scoring system (IBS-SSS)

The 5-item irritable bowel syndrome severity scoring system (IBS-SSS) is a validated and standardized questionnaire for assessing the severity of individual IBS symptoms (Francis et al., 1997). The IBS-SSS ranges from 0 to 500, with higher scores indicating more severe IBS symptoms. Based on the total score, the severity of the symptoms is categorized into remission (<75), mild (76–175), moderate (176–300) and severe (>300). A reduction in the score by at least 50 points is regarded as a clinical improvement (Francis et al., 1997).

#### 2.4.2 Secondary outcome measures

##### 2.4.2.1 Irritable bowel syndrome quality of life (IBS-QoL)

Irritable bowel syndrome quality of life (IBS-QoL) is a validated health-related quality of life questionnaire assessing how the IBS symptoms affect the participants' quality of life (Drossman et al., 2000). Additional to an overall score, IBS-QoL consists of eight subscale scores: dysphoria, interference with activity, body image, health worry, food avoidance, social reaction, sexual, and relationships. Both the overall score and the subscale scores range from 0 to 100 with lower scores indicating poorer quality of life (Drossman et al., 2000). An increase in the score by at least 14 points is regarded as a clinical improvement (Drossman et al., 2007).

##### 2.4.2.2 Hospital anxiety and depression scale (HADS)

Hospital Anxiety and Depression Scale (HADS) is a validated questionnaire developed to identify anxiety and depression in patients admitted to non-psychiatric hospital clinics (Zigmond and Snaith, 1983). The questionnaire includes 14 questions, of which 7 constitute the depression subscale, and the remaining 7 constitute the anxiety subscale. The score of each subscale ranges from 0 to 21 with higher scores indicating more severe anxiety or depression (Zigmond and Snaith, 1983). A cutoff score of ≥8 is recommended for each subscale when screening for depression and anxiety in individuals with IBS (Snijkers et al., 2021).

##### 2.4.2.3 Baseline variables

A study-specific questionnaire was administered at baseline to collect background characteristics (Supplementary file 2). The Rome IV questionnaire with diagnostic criteria for IBS was also administered at baseline (Palsson et al., 2016a). This questionnaire is widely used in IBS research, and has a specificity of 62.7% (Palsson et al., 2016b). To explore the individuals' perceptions of treatment credibility and their expectancy for improvement during the treatment, the six-item Credibility and Expectancy scale (CEQ) was administered at baseline (Devilly and Borkovec, 2000). Each subscale consists of three questions. Different methods for scoring the scale are used (Ponten et al., 2024). In our study, all items were scored on a scale from 1 to 9. The raw total score for each subscale ranging from 3 to 27 for each subscale was used, with higher scores indicating greater treatment credibility and improvement expectancy.

#### 2.4.3 Module adherence and use of message function

Module completion was the primary measure of adherence based on passive logging in the platform. A module was considered completed if the participant had navigated through all pages of a module and spent at least 10 min on it to ensure exposure to the material.

Use of message function was based on passive logging in the platform. The function was considered used if the participant had sent one or more messages during the intervention period.

#### 2.4.4 Treatment experiences

##### 2.4.4.1 Client satisfaction questionnaire (CSQ-8)

Client Satisfaction Questionnaire (CSQ-8) is an 8-item questionnaire that measures how satisfied the participants are with the treatment they have received (Larsen et al., 1979). The total score ranges from 8 to 32 with a higher score indicating a higher level of overall satisfaction (Larsen et al., 1979).

### 2.5 Statistical analysis

#### 2.5.1 Power calculation

At trial registration, the sample size was calculated based on continuous IBS-SSS scores using *t*-test assumptions. Assuming a small effect size (0.25), a two-sided significance level of 5% and 80% power, we calculated that we would need 128 participants in each group. To account for the 42% dropout rate based on previous observations (Berentsen et al., 2025), we planned to enroll 182 participants in each group, giving a total of 728 participants.

Before recruitment was finalized, the sample size calculation was revised to align with the pre-specified primary outcome using the appropriate test for proportions.

This recalculation was based on the primary outcome of IBS symptom responders at 3 months, defined by a clinical cut-off value of ≥50 points for symptom improvement on the IBS-SSS. Based on assumptions of a response rate of 27% in the control group (Bosman et al., 2021) and 55% in each intervention group (estimated from unpublished clinical data), 54 participants per group were required to provide 80% power to detect differences in responder rates between the control group and each intervention group at a two-sided 5% significance level. To account for the anticipated 42% dropout rate (Berentsen et al., 2025), we estimated that we needed 77 participants per group.

Recruitment continued beyond this target to include all eligible participants within the recruitment window. Based on the observed sample size and assumptions used in the revised calculation, the achieved sample size corresponded to >90% power to detect differences in responder rates between groups.

#### 2.5.2 Data analysis

Statistical analyses were conducted according to the registered protocol using R v4.5.0 by the same researcher (CT) who generated the randomization sequence, enrolled participants and assigned interventions. Hence, blinding to treatment allocation during the analysis phase was not feasible.

Descriptive statistics summarized baseline characteristics and clinical outcomes at each time point. Continuous variables are presented as mean ± SD, and categorical variables as n (%). Logistic regression models were fitted to explore predictors of dropout with odds ratio (OR) as effect size estimate. To provide a broad exploration of factors potentially associated with attrition, these exploratory analyses included all available baseline characteristics (randomization group, Rome IV IBS diagnosis, Rome IV IBS subtype, IBS-SSS, credibility score, expectancy score, IBS-QoL overall score, HADS anxiety score, HADS depression score, age, sex, BMI, education, work status, marital status, number of upper endoscopies, number of colonoscopies, extraintestinal comorbidities, duration of gastrointestinal complaints, experienced effect of the FODMAP diet prior to study onset) and post-randomization treatment-related variables (use of message function and percentage of assigned modules completed).

Primary analyses at the 3-month endpoint were conducted on participants with available outcome data. Between-group differences in the primary endpoint of IBS-SSS responder rates at 3 months were analyzed using Fisher's exact test. Responder rates at 6 months were analyzed exploratory for participants with available outcome data. Between-group differences in continuous outcomes at the primary endpoint of 3 months were examined using analysis of covariance (ANCOVA) on all randomized participants with available outcome data, with baseline score included as a covariate, group assignment as a fixed factor and the general patient education group as the reference group. Effect sizes were quantified using partial eta-squared (partial η^2^). Exploratory analyses of within-group changes from baseline to 3 months were assessed using paired *t*-tests, with effect sizes quantified using Cohen's d. Exploratory analyses of longitudinal effects and intention-to-treat estimates were examined using linear mixed models (LMMs) including all randomized participants with at least one measurement. The model included group, time (as a factor), and the group × time interaction as fixed effects, with a random intercept for each participant. Missing data were handled using full information maximum likelihood estimation under the assumption that data were missing at random (MAR).

For treatment experiences (CSQ-8) and adherence, descriptive statistics were used to summarize observed mean and standard deviation per group at 3 months. Between-group differences were analyzed using one-way ANOVA (continuous) and chi-square tests (categorical). Fractional logistic regression was used to examine predictors of module completion, using the same set of baseline and treatment-related variables that were used in the dropout analyses.

## 3 Results

### 3.1 Participant randomization and background characteristics

**Fig. 2:**
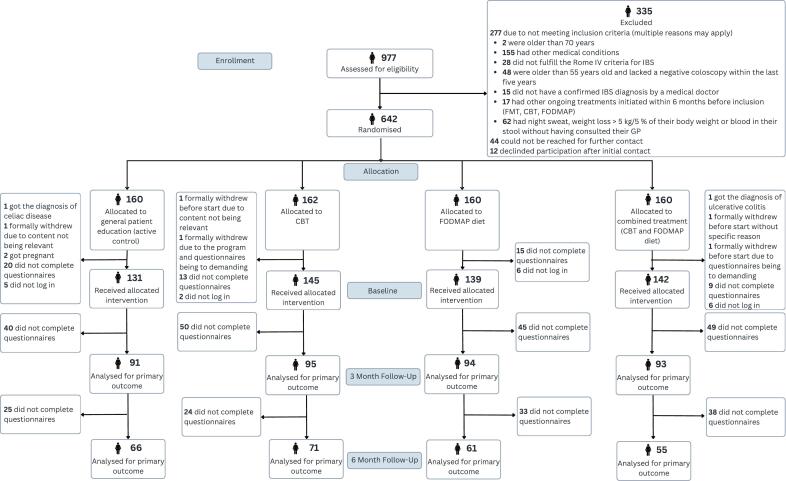
CONSORT flow diagram illustrating participant flow through the study.

A total of 977 people filled out the screening form for assessment of eligibility (Fig. 2). Of the 642 eligible participants that were included and randomized, 557 (87%) completed the baseline questionnaires and were included in the baseline analyses. Of the 557 participants who received their allocated intervention 373 (67%) completed assessments at 3 months and were included in the primary outcome analyses, while 253 (45%) completed assessments at 6 months. Notably, completion of assessments at 6 months should be interpreted conditionally on retention at 3 months, as participants who dropped out at 3 months were withdrawn from the study.

Exploratory dropout analyses at 3 months indicated that a higher number of previous upper endoscopies was associated with higher odds of dropout, whereas having experienced a very large treatment effect of the FODMAP diet prior to recruitment, using the message function and higher module completion were all associated with lower odds of dropout (Supplementary File 3). The associations related to treatment-related variables should be interpreted cautiously, as these are post-randomization measures and may be influenced by individual factors.

At 6 months, fatigue was associated with higher odds of dropout, while a higher number of previous colonoscopies, longer duration of gastrointestinal complaints, IBS with diarrhea, using the message function and higher module completion were all associated with lower odds of dropout (Supplementary File 3).

There were no significant differences in dropout rates between the randomized groups at 3 months (χ^2^(3) = 0.67, *p* = 0.879) or 6 months (χ^2^(3) = 4.73, *p* = 0.193).

The average baseline IBS-SSS score was 295 (SD = 75.4). For the IBS-QoL, the mean baseline score was 48.3 (SD = 20.0). The average baseline scores on the HADS subscales were 9.37 (SD = 4.24) for anxiety and 5.52 (SD = 3.43) for depression. The baseline CEQ scores reflected moderate-high treatment credibility (mean = 18.1 and SD = 4.63) and improvement expectancy (mean = 16.1 and SD = 4.52). Table 3 presents the baseline characteristics of the participants by treatment group and overall.

**Table 3:** Baseline characteristics by treatment group and overall.

	Total *N* = 557	General patient education *N* = 131	CBT *N* = 145	FODMAP diet *N* = 139	Combined CBT and FODMAP diet *N* = 142
Demographic characteristics					
Mean age, years	40.6 (12.5)	38.6 (12.4)	42.2 (12.6)	39.6 (11.9)	41.7 (12.9)
Sex					
*Male*	90 (16.2%)	25 (19.1%)	31 (21.4%)	16 (11.5%)	18 (12.7%)
*Female*	467 (83.8%)	106 (80.9%)	114 (78.6%)	123 (88.5%)	124 (87.3%)
Highest education					
*Elementary school*	20 (3.6%)	3 (2.3%)	5 (3.50%)	7 (5.1%)	5 (3.6%)
*High school*	143 (26.0%)	34 (26.2%)	40 (28.0%)	40 (29.0%)	29 (20.7%)
*College/University*	388 (70.4%)	93 (71.5%)	98 (68.5%)	91 (65.9%)	106 (75.7%)
Employment					
*Student*	55 (10.0%)	15 (11.5%)	8 (5.6)	14 (10.2%)	18 (12.9%)
*Full-time*	318 (57.8%)	82 (63.1%)	74 (51.7%)	78 (56.9%)	84 (60.0%)
*Part-time*	58 (10.5%)	10 (7.7%)	22 (15.4%)	15 (10.9%)	11 (7.9%)
*Disabled/sick leave*	102 (18.5%)	22 (16.9%)	33 (23.1%)	27 (19.7%)	20 (14.3%)
*Retired*	17 (3.1%)	1 (0.8%)	6 (4.2%)	3 (2.2%)	7 (5.0%)
Marital status					
Married	211 (38.3%)	47 (36.2%)	64 (44.8%)	47 (34.1%)	53 (37.9%)
Cohabitant	168 (30.5%)	49 (37.7%)	33 (23.1%)	45 (32.6%)	41 (29.3%)
Single	172 (31.2%)	34 (26.2%)	46 (32.2%)	46 (33.3%)	46 (32.9%)
Diagnosis and Treatment variables					
Previously followed the FODMAP diet	237 (43.0%)	53 (40.8%)	65 (45.1%)	57 (41.3%)	62 (44.6%)
Experienced effect of the FODMAP diet					
*No effect*	26 (11.0%)	6 (11.3%)	7 (10.8%)	6 (10.5%)	7 (11.3%)
*Small effect*	77 (32.5%)	15 (28.3%)	23 (35.4%)	15 (26.3%)	24 (38.7%)
*Medium effect*	94 (39.7%)	23 (43.4%)	24 (36.9%)	27 (47.4%)	20 (32.3%)
*Large effect*	31 (13.1%)	7 (13.2%)	9 (13.8%)	7 (12.3%)	8 (12.9%)
*Very large effect*	9 (3.8%)	2 (3.8%)	2 (3.1%)	2 (3.5%)	3 (4.8%)
Currently following the FODMAP diet	148 (26.9%)	38 (29.2%)	41 (28.5%)	34 (24.6%)	35 (25.2%)
Experienced effect of the FODMAP diet					
*No effect*	3 (2.0%)	1 (2.6%)	0 (0.0%)	1 (2.9%)	1 (2.9%)
*Small effect*	9 (6.1%)	3 (7.9%)	1 (2.4%)	4 (11.8%)	1 (2.9%)
*Medium effect*	60 (40.5%)	14 (36.8%)	16 (39.0%)	14 (41.2%)	16 (45.7%)
*Large effect*	51 (34.5%)	13 (34.2%)	19 (46.3%)	8 (23.5%)	11 (31.4%)
*Very large effect*	25 (16.9%)	7 (18.4%)	5 (12.2%)	7 (20.6%)	6 (17.1%)
Years with GI Complaints	17.9 (12.6)	16.4 (11.1)	20.4 (13.8)	16.5 (11.9)	18.2 (13.1)
Number of upper endoscopies	1.23 (1.5)	1.01 (1.2)	1.50 (1.8)	1.13 (1.4)	1.26 (1.7)
Number of colonoscopies	1.25 (1.3)	1.26 (1.2)	1.39 (1.3)	1.15 (1.2)	1.20 (1.3)
Extraintestinal symptoms					
*Muscle pain (fibromyalgia)*	106 (20.4%)	17 (14.2%)	25 (18.2%)	29 (22.0%)	35 (26.9%)
*Headache*	104 (20.0%)	23 (19.2%)	26 (19.0%)	25 (18.9%)	30 (23.1%)
*Genital discomfort*	40 (7.7%)	12 (10.0%)	10 (7.3%)	12 (9.1%)	6 (4.6%)
*Chronic fatigue*	131 (25.2%)	28 (23.3%)	40 (29.2%)	37 (28.0%)	26 (20.0%)
*Depression*	32 (6.2%)	8 (6.7%)	7 (5.11%)	8 (6.1%)	9 (6.9%)
*Sleep problems*	106 (20.4%)	32 (26.7%)	29 (21.2%)	21 (15.9%)	24 (18.5%)
Rome IV IBS positive	429 (77.7%)	100 (77.5%)	112 (78.3%)	102 (73.4%)	115 (81.6%)
*Constipation subtype*	131 (23.8%)	29 (22.5%)	37 (26.1%)	35 (25.2%)	30 (21.4%)
*Diarrhea subtype*	189 (34.4%)	48 (37.2%)	44 (31.0%)	45 (32.4%)	52 (37.1%)
*Mixed subtype*	220 (40.0%)	50 (38.8%)	57 (40.1%)	58 (41.7%)	55 (39.3%)
*Unclassified subtype*	10 (1.8%)	2 (1.6%)	4 (2.8%)	1 (0.7%)	3 (2.1%)
Baseline scores					
IBS Symptom Severity Score	295 (75.4)	300 (78.5)	300 (76.2)	290 (75.9)	290 (71.4)
Overall IBS-Quality of Life Score	48.3 (20.0)	49.8 (21.1)	46.0 (20.4)	48.2 (18.1)	49.2 (20.2)
HADS-Anxiety Score	9.37 (4.2)	9.35 (4.4)	9.41 (4.3)	9.95 (4.2)	8.76 (4.1)
HADS-Depression Score	5.52 (3.4)	5.61 (3.6)	5.62 (3.3)	5.92 (3.4)	4.95 (3.4)
Credibility Sum Score	18.1 (4.6)	18.5 (4.8)	18.0 (4.8)	18.3 (4.4)	17.5 (4.5)
Expectancy Sum Score	16.1 (4.5)	16.4 (4.7)	16.2 (4.8)	16.1 (4.1)	15.8 (4.1)

Note: All groups received internet-delivered general patient education. CBT-based and FODMAP diet modules were provided as additional components as indicated. Continuous variables are presented as mean ± standard deviation, and categorical variables as n (%); CBT = Cognitive behavioral therapy; FODMAP = fermentable oligosaccharides, disaccharides, monosaccharides, and polyols; GI = gastrointestinal; IBS = irritable bowel syndrome; HADS = Hospital Anxiety and Depression Scale.

### 3.2 Module adherence and use of message function

Across all groups, the first three modules (the general patient education modules) were the most frequently completed at 3 months (Table 4). Specifically, 46 out of 131 participants (35.1%) in the general patient education group, 59 out of 145 (40.7%) in the CBT group, 50 out of 139 (36.0%) in the FODMAP diet group, and 49 out of 142 (34.5%) in the combined CBT and FODMAP diet group completed all the first three modules (Table 4).

**Table 4:** Completion of the first three modules per group (3 months).

Group	N	Gastroenterology N (%)	Physiotherapy N (%)	General lifestyle N (%)	Completion of all three modules N (%)
General patient education	131	70 (53.4%)	63 (48.1%)	52 (39.7%)	46 (35.1%)
CBT	145	88 (60.7%)	77 (53.1%)	65 (44.8%)	59 (40.7%)
FODMAP diet	139	75 (54.0%)	60 (43.2%)	54 (38.8%)	50 (36.0%)
Combined CBT and FODMAP diet	142	77 (54.2%)	65 (45.8%)	53 (37.3%)	49 (34.5%)

Note: Adherence is based on logged data and includes all participants who accessed their assigned intervention. Adherence to a module was considered if the participant had navigated through all pages of a module and spent at least 10 min on it. CBT = Cognitive behavioral therapy; FODMAP = fermentable oligosaccharides, disaccharides, monosaccharides, and polyols.

In contrast, adherence to the content-specific modules was lower (Table 5). Only 29 out of 145 participants (20.0%) completed the CBT-based module in the CBT group, 20 out of 139 (14.4%) completed the FODMAP diet module in the FODMAP diet group, and 14 out of 142 (9.9%) completed both the CBT and FODMAP diet modules in the combined group (Table 5). Use of the message function (OR (95% CI) = 3.39 (2.16 to 5.38), *p* < 0.001) and higher baseline credibility (OR (95% CI) = 1.06 (1.00 to 1.12), *p* = 0.036) were associated with higher percentage module completion at 3 months.

**Table 5:** Completion of the intervention modules per group (3 months).

Group	N	CBT N (%)	FODMAP diet N (%)	Both CBT and FODMAP N (%)
General patient education	131	Not available	Not available	Not available
CBT	145	29 (20.0%)	Not available	Not available
FODMAP diet	139	Not available	20 (14.4%)	Not available
Combined CBT and FODMAP diet	142	35 (24.6%)	19 (13.4%)	14 (9.9%)

Note: Adherence is based on logged data and includes all participants who accessed their assigned intervention. Adherence to a module was considered if the participant had navigated through all pages of a module and spent at least 10 min on it. “Not available” indicate that the module was not available for that group. CBT = Cognitive behavioral therapy; FODMAP = fermentable oligosaccharides, disaccharides, monosaccharides, and polyols.

Although adherence increased at 6 months, the overall pattern remained unchanged (Supplementary file 4). The first three modules continued to be the most frequently completed across all groups, while adherence to the content-specific modules showed a moderate increase. Completing a higher percentage of modules at 3 months was strongly associated with higher percentage of module completion at 6 months (OR (95% CI) = 210.0 (87.4 to 554.0), p < 0.001).

Use of the messaging function was relatively similar across groups. Overall, 154 out of 557 participants (27.6%) used the messaging feature during the intervention. Specifically, 38 out of 131 participants (29.0%) in the general patient education group, 35 out of 145 (24.1%) in the CBT group, 42 out of 139 (30.2%) in the FODMAP diet group, and 39 out of 142 (27.5%) in the combined CBT and FODMAP diet group used the message function once or more. A chi-square test indicated no significant difference in messaging use between groups (χ^2^(3) = 1.48, *p* = 0.688), and the effect size was very small (Cramer's V = 0.051), suggesting similar use across interventions.

### 3.3 Clinical outcomes

For IBS-SSS, clinical improvement at 3 months was observed in 41 out of 91 participants (45.1%) in the general patient education group, 42 out of 95 participants (44.2%) in the CBT group, 40 out of 94 participants (42.6%) in the FODMAP diet group, and 41 out of 93 participants (44.1%) in the combined CBT and FODMAP diet group (Table 6). No statistically significant difference between groups in the number of participants who had experienced clinical improvement at 3 months were observed (OR (95% CI) = 0.97 (0.54 to 1.72), *p* = 0.989). Across treatment groups, participants who experienced clinical improvement (*n* = 164) showed a mean IBS-SSS reduction of 113.9 points (95% CI: 105.2 to 122.7) corresponding to a 36% reduction (95% CI: 33.2% to 38.8%) (Supplementary file 5). Participants with no clinical change (*n* = 157) had a mean reduction of 2.3 points (95% CI: −6.8 to 2.1) corresponding to a 0.8% reduction (95% CI: −2.4% to 0.8%), whereas participants who experienced clinical deterioration (*n* = 52) showed a mean increase of 82.7 points (95% CI: 74.6 to 90.9) corresponding to a 31.6% increase (95% CI: 28.5 to 34.8%).

Consistent with the findings for IBS-SSS, analyses of IBS-QoL overall score and subscales revealed no statistically significant difference between groups in the number of participants that experienced clinical improvement at 3 months (Table 6). Similarly, analyses of six-month data did not indicate any statistically significant difference in the number of participants who experienced clinical improvement (Supplementary file 6).

Exploratory analyses of IBS-SSS as a continuous measure showed no between-group change were found after adjusting for baseline scores (*p* = 0.947, partial η^2^ = 0.001)(Table 7). Analyses of IBS-QoL and HADS were consistent with this finding (all *p* > 0.05, partial η^2^ < 0.02) (Table 7). Across all groups, moderate within-group treatment effects were observed at 3 months for IBS-SSS, IBS-QoL and HADS (Table 7).

**Table 6:** Meaningful clinical change after 3 months.

Treatment Group	N	Clinically Deteriorated	No clinical Change	Clinically Improved	Between-group *p*-value clinically improvement	Between-group OR (95% CI)
IBS-SSS						
General patient education	91	10 (11%)	40 (44%)	41 (45.1%)	0.989	0.97 [0.54, 1.72]
CBT	95	14 (14.7%)	39 (41.1%)	42 (44.2%)		
FODMAP diet	94	16 (17%)	38 (40.4%)	40 (42.6%)		
Combined CBT and FODMAP diet	93	12 (12.9%)	40 (43%)	41 (44.1%)		

IBS-QoL Overall Score						
General patient education	90	6 (6.7%)	67 (74.4%)	17 (18.9%)	0.729	1.15 [0.56, 2.36]
CBT	95	3 (3.2%)	72 (75.8%)	20 (21.1%)		
FODMAP diet	93	4 (4.3%)	70 (75.3%)	19 (20.4%)		
Combined CBT and FODMAP diet	94	1 (1.1%)	69 (73.4%)	24 (25.5%)		

IBS-QoL Subscales						
*Body Image Score*						
General patient education	90	7 (7.8%)	66 (73.3%)	17 (18.9%)	0.596	1.00 [0.48, 2.10]
CBT	95	5 (5.3%)	72 (75.8%)	18 (18.9%)		
FODMAP diet	93	5 (5.4%)	64 (68.8%)	24 (25.8%)		
Combined CBT and FODMAP diet	94	9 (9.6%)	63 (67%)	22 (23.4%)		

*Dysphoria score*						
General patient education	90	9 (10%)	55 (61.1%)	26 (28.9%)	0.489	1.14 [0.61, 2.13]
CBT	95	4 (4.2%)	61 (64.2%)	30 (31.6%)		
FODMAP diet	92	6 (6.5%)	55 (59.8%)	31 (33.7%)		
Combined CBT and FODMAP diet	94	6 (6.4%)	51 (54.3%)	37 (39.4%)		

*Food Avoidance Score*						
General patient education	90	16 (17.8%)	48 (53.3%)	26 (28.9%)	0.097	0.79 [0.41, 1.51]
CBT	95	13 (13.7%)	59 (62.1%)	23 (24.2%)		
FODMAP diet	92	18 (19.6%)	54 (58.7%)	20 (21.7%)		
Combined CBT and FODMAP diet	94	15 (16%)	44 (46.8%)	35 (37.2%)		

*Health Worry Score*						
General patient education	90	10 (11.1%)	46 (51.1%)	34 (37.8%)	0.992	1.00 [0.55, 1.82]
CBT	95	16 (16.8%)	43 (45.3%)	36 (37.9%)		
FODMAP diet	92	12 (13%)	47 (51.1%)	33 (35.9%)		
Combined CBT and FODMAP diet	94	13 (13.8%)	46 (48.9%)	35 (37.2%)		

*Interference with Activity Score*						
General patient education	90	6 (6.7%)	55 (61.1%)	29 (32.2%)	0.685	1.23 [0.67, 2.25]
CBT	95	6 (6.3%)	54 (56.8%)	35 (36.8%)		
FODMAP diet	93	10 (10.8%)	54 (58.1%)	29 (31.2%)		
Combined CBT and FODMAP diet	94	10 (10.6%)	48 (51.1%)	36 (38.3%)		

*Relationships Score*						
General patient education	90	13 (14.4%)	49 (54.4%)	28 (31.1%)	0.727	1.07 [0.58, 1.99]
CBT	95	17 (17.9%)	47 (49.5%)	31 (32.6%)		
FODMAP diet	93	11 (11.8%)	58 (62.4%)	24 (25.8%)		
Combined CBT and FODMAP diet	94	13 (13.8%)	55 (58.5%)	26 (27.7%)		

*Social Reaction Score*						
General patient education	90	10 (11.1%)	61 (67.8%)	19 (21.1%)	0.507	1.00 [0.49, 2.02]
CBT	95	16 (16.8%)	59 (62.1%)	20 (21.1%)		
FODMAP diet	93	8 (8.6%)	59 (63.4%)	26 (28%)		
Combined CBT and FODMAP diet	94	10 (10.6%)	66 (70.2%)	18 (19.1%)		

*Sexual Function Score*						
General patient education	89	10 (11.2%)	58 (65.2%)	21 (23.6%)	0.491	0.71 [0.34, 1.45]
CBT	95	8 (8.4%)	70 (73.7%)	17 (17.9%)		
FODMAP diet	92	8 (8.7%)	65 (70.7%)	19 (20.7%)		
Combined CBT and FODMAP diet	93	9 (9.7%)	59 (63.4%)	25 (26.9%)		

Note: IBS-SSS is presented first, followed by the IBS-QoL overall score and IBS-QoL subscales (italics). Number of participants categorized as Clinically Deteriorated, No clinical Change or Clinically Improved are calculated using cut-offs that are regarded as clinical meaningful and presented as n (%); IBS-SSS threshold = 50 points (lower score indicate improvement); IBS-QoL subscales threshold = 14 points (higher score indicate improvement); Between-group differences after three months were analyzed using Fisher's exact test with effect sizes reported as Cramer's V (95% CI); IBS-SSS = Irritable Bowel Syndrome Symptom Severity Score; IBS-QoL = Irritable Bowel Syndrome Quality of Life; CBT = Cognitive behavioral therapy; FODMAP = fermentable oligosaccharides, disaccharides, monosaccharides, and polyols.

**Table 7:** Observed means, within-group effect sizes and results from ANCOVA after three months.

	Baseline		3 Months		Within-Group Effect Size 0-3 m			ANCOVA Results		
Treatment Group	N	Mean (SD)	N	Mean (SD)	Change (SD)	Cohens d (95% CI)	P-value	B (95% CI)	p-value	Effect size (partial η^2^)
IBS-SSS										
General patient education	131	300.1 (78.5)	91	252.5 (96.7)	−42.6 (86.3)	−0.49 (−0.71, −0.28)	<0.001	Reference	0.947	0.001
CBT	145	300.3 (76.2)	95	262.1 (98.7)	−36.9 (79.5)	−0.46 (−0.68, −0.25)	<0.001	6.99 (−16.19, 30.18)		
FODMAP diet	139	290.4 (75.9)	94	254.0 (94.3)	−39.8 (88.5)	−0.45 (−0.66, −0.24)	<0.001	2.41 (−20.83, 25.65)		
Combined CBT and FODMAP diet	142	290.3 (71.4)	93	252.9 (88.5)	−39.0 (81.8)	−0.48 (−0.69, −0.26)	<0.001	2.53 (−20.77, 25.84)		

IBS-QoL Overall Score										
General patient education	130	49.8 (21.1)	90	55.4 (19.8)	5.2 (14.0)	0.38 (0.16, 0.59)	<0.001	Reference	0.638	0.005
CBT	144	46.0 (20.4)	95	51.3 (21.2)	5.9 (11.5)	0.51 (0.30, 0.72)	<0.001	−0.38 (−4.04, 3.28)		
FODMAP diet	139	48.2 (18.1)	93	54.0 (19.5)	5.6 (11.4)	0.49 (0.28, 0.71)	<0.001	−0.00 (−3.67, 3.66)		
Combined CBT and FODMAP diet	141	49.2 (20.2)	94	54.7 (21.5)	7.7 (15.9)	0.49 (0.27, 0.70)	<0.001	1.80 (−1.86, 5.46)		

IBS-QoL Subscales										
*Body Image Score*										
General patient education	130	46.1 (24.9)	90	49.9 (23.7)	3.5 (17.1)	0.21 (−0.00, 0.42)	0.052	Reference	0.594	0.005
CBT	144	42.7 (21.8)	95	47.1 (23.6)	4.4 (13.4)	0.33 (0.12, 0.54)	0.002	0.28 (−4.10, 4.66)		
FODMAP diet	139	42.7 (19.5)	93	50.1 (24.1)	6.7 (14.9)	0.45 (0.23, 0.66)	<0.001	2.66 (−1.74, 7.07)		
Combined CBT and FODMAP diet	141	44.5 (21.9)	94	49.1 (25.4)	5.8 (16.6)	0.35 (0.14, 0.56)	0.001	1.75 (−2.64, 6.14)		

*Dysphoria Score*										
General patient education	130	49.6 (25.9)	90	56.5 (25.0)	6.8 (19.3)	0.36 (0.14, 0.57)	0.001	Reference	0.357	0.009
CBT	144	43.7 (24.5)	95	53.2 (25.7)	9.3 (13.9)	0.67 (0.45, 0.89)	<0.001	1.07 (−3.67, 5.82)		
FODMAP diet	139	46.5 (23.7)	92	55.0 (25.7)	7.5 (15.5)	0.48 (0.27, 0.70)	<0.001	0.13 (−4.64, 4.90)		
Combined CBT and FODMAP diet	141	49.5 (25.9)	94	58.0 (24.6)	11.4 (20.6)	0.55 (0.33, 0.77)	<0.001	3.81 (−0.94, 8.56)		

*Food Avoidance Score*										
General patient education	130	32.1 (24.7)	90	35.6 (24.9)	3.4 (19.8)	0.17 (−0.04, 0.38)	0.105	Reference	0.073	0.019
CBT	144	30.2 (24.2)	95	30.5 (22.3)	3.6 (16.3)	0.22 (0.02, 0.42)	0.034	−1.57 (−6.81, 3.66)		
FODMAP diet	139	29.6 (24.9)	92	31.6 (24.6)	0.9 (18.4)	0.05 (−0.16, 0.25)	0.654	−3.06 (−8.32, 2.19)		
Combined CBT and FODMAP diet	141	29.1 (23.1)	94	37.5 (24.1)	7.9 (23.4)	0.34 (0.13, 0.54)	0.002	3.60 (−1.63, 8.84)		

*Health Worry Score*										
General patient education	130	51.8 (26.5)	90	60.4 (22.6)	6.9 (18.8)	0.36 (0.15, 0.58)	<0.001	Reference	0.793	0.003
CBT	144	51.4 (24.7)	95	57.2 (25.0)	5.8 (20.5)	0.28 (0.08, 0.49)	0.007	−1.83 (−6.85, 3.19)		
FODMAP diet	139	51.0 (21.3)	92	58.9 (20.6)	6.5 (17.7)	0.37 (0.16, 0.58)	<0.001	−0.75 (−5.81, 4.30)		
Combined CBT and FODMAP diet	141	53.1 (24.0)	94	59.9 (24.7)	8.1 (20.8)	0.39 (0.18, 0.60)	<0.001	0.61 (−4.42, 5.64)		

*Interference with Activity Score*										
General patient education	130	47.0 (24.3)	90	54.2 (22.8)	6.7 (17.3)	0.38 (0.17, 0.60)	<0.001	Reference	0.815	0.003
CBT	144	42.0 (24.2)	95	48.2 (24.0)	7.9 (14.3)	0.55 (0.33, 0.77)	<0.001	−0.52 (−4.99, 3.96)		
FODMAP diet	139	47.0 (23.2)	93	51.2 (23.0)	5.6 (14.7)	0.38 (0.17, 0.59)	<0.001	−1.56 (−6.03, 2.92)		
Combined CBT and FODMAP diet	141	45.4 (23.8)	94	51.0 (25.3)	8.4 (19.0)	0.44 (0.23, 0.65)	<0.001	0.55 (−3.92, 5.02)		

*Relationship Score*										
General patient education	130	62.9 (25.3)	90	67.5 (22.3)	4.8 (17.4)	0.28 (0.07, 0.49)	0.010	Reference	0.794	0.003
CBT	144	58.5 (25.7)	95	62.9 (25.9)	4.0 (17.7)	0.23 (0.02, 0.43)	0.028	−1.92 (−6.44, 2.61)		
FODMAP diet	139	62.7 (23.0)	93	66.6 (22.6)	3.5 (14.4)	0.25 (0.04, 0.45)	0.020	−1.16 (−5.70, 3.38)		
Combined CBT and FODMAP diet	141	62.3 (25.3)	94	64.5 (23.8)	6.1 (19.5)	0.31 (0.11, 0.52)	0.003	0.04 (−4.50, 4.57)		

*Social Relation Score*										
General patient education	130	57.6 (25.3)	90	61.7 (22.6)	2.6 (16.3)	0.16 (−0.05, 0.37)	0.129	Reference	0.450	0.007
CBT	144	56.1 (25.2)	95	57.7 (24.1)	1.8 (17.6)	0.10 (−0.10, 0.31)	0.310	−1.83 (−6.14, 2.48)		
FODMAP diet	139	57.0 (23.5)	93	62.2 (21.1)	4.9 (16.2)	0.30 (0.09, 0.51)	0.004	1.70 (−2.62, 6.03)		
Combined CBT and FODMAP diet	141	58.2 (24.6)	94	59.6 (23.8)	3.3 (17.7)	0.18 (−0.02, 0.39)	0.078	−0.25 (−4.56, 4.07)		

*Sexual score*										
General patient education	129	56.0 (32.0)	89	57.9 (29.1)	3.5 (23.1)	0.15 (−0.06, 0.36)	0.155	Reference	0.526	0.006
CBT	144	52.7 (29.7)	95	54.3 (30.5)	2.0 (19.6)	0.10 (−0.10, 0.30)	0.328	−2.08 (−7.72, 3.56)		
FODMAP diet	139	54.0 (27.5)	92	59.6 (28.7)	5.0 (21.5)	0.23 (0.03, 0.44)	0.027	1.59 (−4.09, 7.27)		
Combined CBT and FODMAP diet	141	56.5 (31.0)	93	59.0 (29.0)	5.2 (20.5)	0.26 (0.05, 0.46)	0.015	1.57 (−4.10, 7.23)		

Anxiety Score (HADS-A)										
General patient education	130	9.4 (4.4)	90	9.1 (4.3)	−0.0 (3.2)	−0.01 (−0.21, 0.20)	0.948	Reference	0.177	0.013
CBT	143	9.4 (4.3)	95	9.3 (4.4)	−0.3 (3.6)	−0.09 (−0.29, 0.11)	0.385	−0.15 (−0.98, 0.67)		
FODMAP diet	138	9.9 (4.2)	92	9.0 (4.1)	−0.5 (2.7)	−0.17 (−0.38, 0.03)	0.097	−0.34 (−1.18, 0.49)		
Combined CBT and FODMAP diet	140	8.8 (4.1)	95	8.3 (3.8)	−0.9 (2.9)	−0.33 (−0.54, −0.12)	0.002	−0.88 (−1.70, −0.05)		

Depression Score (HADS-D)										
General patient education	130	5.6 (3.6)	90	5.5 (3.9)	0.3 (2.9)	0.09 (−0.12, 0.30)	0.401	Reference	0.316	0.010
CBT	143	5.6 (3.2)	95	5.2 (3.5)	−0.5 (2.5)	−0.18 (−0.38, 0.02)	0.080	−0.60 (−1.33, 0.13)		
FODMAP diet	138	5.9 (3.4)	92	5.2 (3.5)	−0.4 (2.7)	−0.14 (−0.35, 0.06)	0.180	−0.54 (−1.27, 0.19)		
Combined CBT and FODMAP diet	140	5.0 (3.4)	95	4.8 (3.0)	−0.3 (2.6)	−0.10 (−0.30, 0.10)	0.338	−0.58 (−1.30, 0.15)		

Note: IBS-SSS is presented first, followed by the IBS-QoL overall score, IBS-QoL subscales (italics), HADS-A and HADS-D. CBT = cognitive behavioral therapy; FODMAP = fermentable oligosaccharides, disaccharides, monosaccharides, and polyols; IBS-SSS = Irritable Bowel Syndrome Symptom Severity Score; QoL = Quality of Life; HADS = Hospital Anxiety and Depression Scale; N = observed number of participants with data at each timepoint; Change (SD) = Mean change from baseline with SD of the change scores; Cohen's d: 0.2 = small, 0.5 = medium, 0.8 = large effect size (Negative Cohen's d indicates improvement for IBS-SSS and HADS; positive indicates improvement for IBS-QoL); Beta estimates represent difference from reference group (General patient education) adjusted for baseline scores; P-value from Type III ANOVA F-test for overall treatment effect; Partial eta squared: 0.01 = small, 0.06 = medium, 0.14 = large effect.

Exploratory analyses across all three timepoints showed similar results with no consistent between-group differences in the intention-to-treat population, although within-group changes were observed (Supplementary file 7). Some trends of between-group differences emerged in IBS-QoL subscales at 6 months, but these finding should be interpreted as exploratory.

### 3.4 Treatment satisfaction

Treatment satisfaction, as measured by CSQ-8, was generally high across all groups. The average score (SD) was 22.7 (4.1) in the general patient education group, 23.2 (4.0) in the CBT group, 22.6 (4.2) in the FODMAP diet group, and 23.1 (4.3) in the combined CBT and FODMAP diet group. No statistically significant differences between the groups were observed (*p* = 0.677, partial η^2^ (95% CI) = 0.004) (Supplementary file 8).

## 4 Discussion

In this randomized controlled trial, we evaluated the effectiveness of a CBT-based module, a FODMAP diet module, or their combination, when added to internet-delivered general patient education, compared to general patient education alone. Our primary finding was similar IBS-SSS responder rates at 3 months, indicating no additional benefit from the content-specific modules. However, adherence patterns indicated that participants were more likely to complete the general education modules than the content-specific modules at 3 months. Consequently, the lack of between-group effect should be interpreted alongside these adherence patterns.

Consistent with previous studies (Wallen et al., 2025; Ljotsson et al., 2011; Pathipati et al., 2024; Carbone et al., 2022; Dimidi et al., 2023), moderate within-group improvements were observed across all intervention arms, and longitudinal analyses indicated that improvements were maintained across all groups at 6 months. However, the primary objective of the trial was to evaluate between-group effects. As no significant differences were found, we consider potential explanations and implications for future research.

### 4.1 Adherence

Comparable adherence to the initial general patient education modules, combined with lower adherence to the content-specific modules at 3 months, limited the differential exposure between the study groups. Consequently, the observed adherence patterns complicate the interpretation of the effectiveness across study groups and may partly explain the smaller treatment effects observed compared to previous research.

Previous studies of app-delivered FODMAP diet interventions have shown symptom improvement in 63% to 71% of participants, supported by adherence rates of 94% in both studies (Carbone et al., 2022; Dimidi et al., 2023). Similarly, internet-delivered CBT have demonstrated treatment effects beyond ours, also with high adherence of 70.2% to 88.8% (Wallen et al., 2025; Ljotsson et al., 2011). However, a real-world study of self-guided internet-delivered CBT reported adherence to be 38% for half of the program and 19% for the full program, which aligns more closely with our results (Pathipati et al., 2024). Consistent with our findings, they still observed statistically significant treatment effects regardless of the low adherence, although they did not report the effect size (Pathipati et al., 2024).

Several factors may have contributed to the higher adherence observed for the general patient education modules compared with the content-specific modules. Although the program was accessible across digital devices, user interface varied and data on device use were not available. This may be relevant, as user behavior differs between desktop and mobile interfaces (Nielsen and Pernice, 2010; Lagun et al., 2014). The program was originally designed for desktop browsers, where the content-specific modules appeared furthest to the right, whereas these modules were located at the bottom of the screen on smartphones. These differences in user interface may have influenced navigation patterns and the order in which modules were accessed. In addition, the sequential numbering of modules likely encouraged participants to begin with the initial modules and proceed in order.

Beyond user interface, the perceived personal relevance of the content and participants early psychological response to the program may have shaped different usage paths (Christensen and Mackinnon, 2006; Nomeikaite et al., 2023; Ritterband et al., 2009; Smith et al., 2025; Short et al., 2015). In this context, differentiation between dropouts and attainers may be relevant (Martinez, 2003). Attainers may have experienced sufficient benefit from the initial modules, reducing motivation to continue with the additional intervention content, whereas dropouts may have experienced unmet expectations that contributed to early disengagement (Christensen and Mackinnon, 2006; Nomeikaite et al., 2023; Ritterband et al., 2009).

Moreover, the content-specific modules required greater time investment and active behavior change than the general patient education modules. This unequal treatment dose and intervention burden may have contributed to low content-specific adherence and further reduced differential exposure, thereby limiting ability to assess treatment fidelity (Bellg et al., 2004). Treatment burden and treatment fatigue are identified as important barriers to adherence in behavioral medicine, while lack of time is highlighted as a key barrier to engagement in internet-delivered interventions (Nomeikaite et al., 2023; Bryan et al., 2015; Jardine et al., 2024). Although the program was intended to be completed over three months, the observed adherence patterns suggest that a longer timeframe may have been required for participants to fully engage with the content-specific modules.

Importantly, adherence in the present study was assessed only at the module level. While number of modules completed is a commonly used metric of adherence, this does not infer actual behavior adherence (Bellg et al., 2004; Sieverink et al., 2017). Consequently, this limits our ability to determine whether the content-specific modules were adequately delivered, received, and enacted, thereby constraining firm conclusions regarding their effectiveness (Bellg et al., 2004).

Together, these considerations underscore the importance of interpreting the findings of internet-delivered interventions for IBS in a broader context, particularly when evaluating the relative effectiveness of different configurations.

### 4.2 Choice of control group

Designing an optimal control condition in non-pharmacological trials for IBS is challenging, and our study add to the literature highlighting these challenges (Amsallem et al., 2021). In our study, we aimed at providing the participants in the control group a credible and ethically acceptable comparator, under the assumption that the interventions would provide additive benefits beyond this baseline. However, the general patient education group constituted a strong active control intervention, including structured education, behavioral advice, and access to dietitian support.

When designing the study, we anticipated that including information about IBS and practical strategies for implementing recommended first-line management would allowed us to account for the potential effects of symptom explanation, reassurance, and enhanced self-efficacy, previously suggested to play a critical role in symptom management (Tayama et al., 2024; Boyce et al., 2003; Cuijpers et al., 2019; Amsallem et al., 2021). In addition, access to personalized guidance was intended to account for the effect of therapeutic support, which has been demonstrated to be a potent component of non-specific effects in IBS treatment (Kaptchuk et al., 2008).

Considering these factors, and the low adherence to the content-specific modules, this comprehensive education package and personalized guidance likely functioned as a substantive intervention itself, potentially masking additive effects of the CBT-based and FODMAP diet modules. Consistent with this, the responder rate observed in the control group (45.1%) was substantially higher than what we had assumed in the sample size calculation (27%). As the sample size calculation was based on pharmacological placebo response rates, this mismatch with our strong control group likely reduced the contrast between groups and limited the ability to detect incremental effects of the content-specific modules.

In contrast to our study, a recent randomized controlled trial evaluating a multidisciplinary internet-delivered intervention for patients with IBS reported between-group improvements compared to their control group (Tayama et al., 2024). However, their control group received no pharmacological or non-pharmacological treatment, representing a more passive comparator (Tayama et al., 2024). While such designs may increase the likelihood of detecting between-group effects, they offer limited insight into effects of the specific elements. In comparison, our use of an active control condition provides a more stringent test of intervention efficacy. Our results add to the body of literature highlighting the importance of carefully designing control groups and for transparent reporting to facilitate meaningful comparisons across trials. Future research should prioritize methodological clarity in control group design to strengthen the evidence base and inform clinical decision-making.

### 4.3 Choice of primary outcome

Although no significant between-group differences were observed on the primary outcome of global IBS symptoms, data on medication use or dietary variation were not collected. Unmeasured differences in these factors across groups could have affected symptoms and potentially blurred potential treatment effects. To understand treatment effects beyond symptom scores, the food avoidance subscale of the IBS-QoL offers useful information. Earlier research shows that IBS patients with high food avoidance often report more severe symptoms, poorer quality of life, and increased nutritional risk (Nielsen and Pernice, 2010). Reducing food avoidance is therefore an important outcome, as it reflects better quality of life and reduced nutritional risk.

In our study, the largest within-group improvement in the food avoidance subscale was observed in the combined CBT and FODMAP group. Notably, this should be interpreted in the context of low adherence to the content-specific modules. While this observation does not indicate a clear additive treatment effect, it may suggest that the combination of the psychological strategies provided in the CBT-based module and the structured dietary guidance provided in the FODMAP diet module has the potential to influence food-related quality of life.

The CBT-based module provided tools to relate differently to the symptoms and expose them to foods they would normally avoid. This may make the FODMAP reintroduction phase easier and lead to earlier expansion of the diet. Such changes can improve food-related quality of life independent of global IBS symptoms. Because we did not assess actual diet registrations, we cannot confirm this mechanism. Also, our study was not powered to detect differences in the subscale analyses and findings should be interpreted as exploratory.

Overall, our findings suggest that global IBS symptom scores alone may not capture the benefits of integrated treatment. Future studies should include outcomes that capture coping, behavioral flexibility, and food-related quality of life.

### 4.4 Limitations

In the interpretation of the results from this study, several limitations should be considered. First, the study dropout was high with 33% drop-out at 3 months and 55% drop-out at 6 months. Although dropout rates did not differ significantly between groups, attrition was associated with both baseline and post-randomization treatment-related variables. This selective retention may have resulted in a follow-up sample with more motivated or responsive participants, potentially overstating within-group improvements and masking potential between-group differences in the primary outcome. Second, the primary analyses of continuous outcomes were based on complete cases using ANCOVA, with the 3-month follow-up defined a priori as the primary endpoint. To assess the robustness of the findings, we additionally conducted intention-to-treat analyses using linear mixed models with full information maximum likelihood imputation under the assumption of data missing at random. Although dropout was associated with observed variables, the assumption that data was missing at random remains untestable. If missingness were additionally related to unobserved variables, estimates from the linear mixed models could still be biased. Third, the study was unblinded, which may introduce performance bias. The randomization sequence was not concealed, but adherence to the sequence was carefully monitored and participant enrollment was conducted with the assistance of an independent study nurse, minimizing the risk of selection and allocation bias. Outcomes were assessed using standardized digital self-report questionnaires, reducing the likelihood of detection bias. All analyses were conducted according to the preregistered protocol, which mitigates the risk of analysis bias. Nonetheless, reliance on self-reported outcomes may still introduce reporting and expectancy biases. Fifth, as this was a fully digital intervention, we had limited control over concurrent behaviors or treatments outside the program. We did not collect ongoing data on medication use, physical activity changes, further psychological therapies, or detailed dietary intake, and could not adjust for potential confounding factors. While the inclusion of an active control group strengthens internal validity, the absence of a passive comparator (treatment as usual or a wait-list control) limits our ability to determine whether the observed effects would occur in the absence of treatment, particularly given the well-documented fluctuation of IBS symptoms over time (Mearin et al., 2004; Yadav et al., 2021). On the other hand, the internal validity may have been limited by the differences in content complexity and intervention dose between groups, as the number of modules varied across groups. These differences in participant burden may have influenced engagement and adherence. Moreover, adherence was measured passively as module completion, without assessing active engagement. In addition, the absence of a systematic evaluation of barriers to participation limits our ability to draw hard conclusions on reasons for the low adherence to the content-specific modules.

### 4.5 Conclusion and implications

In this four-armed randomized controlled trial, all intervention groups demonstrated clinically and statistically meaningful improvements in IBS symptoms and IBS-related quality of life at 3 months, with minimal clinician involvement. Although no additional benefit of the content-specific modules was observed, this finding should be interpreted in the context of the low adherence to these modules.

Taken together, the results highlight the value of low-intensity internet-delivered interventions as a scalable approach for IBS management within resource-constrained health systems, while emphasizing the importance of addressing adherence and engagement in future research.

However, internet-delivered interventions may not be suitable for all patients. Future research should focus on identifying patient-related predictors of response to internet-delivered interventions, to improve personalization and guide clinical decision-making. Further work is needed to determine which patients may benefit from the different intervention components or from varying levels of clinician guidance to optimize outcomes and guide program optimization.

## Abbreviations

IBS irritable bowel syndrome

FODMAP fermentable oligosaccharides, disaccharides, monosaccharides, and polyols

CBT Cognitive behavioral therapy

IBS-SSS IBS severity scale

CONSORT Consolidated Standards of Reporting Trials

HUH Haukeland University Hospital

RCT randomized controlled trial

IBS-QoL Irritable bowel syndrome quality of life

HADS Hospital Anxiety and Depression Scale

CEQ Credibility/Expectancy scale

CSQ-8 Client Satisfaction Questionnaire

ANCOVA analysis of covariance

## Ethics

This study was reviewed and approved by Regional Committees for Medical and Health Research Ethics Western Norway, ID # 630038. The participants provided their written informed consent to participate in this study.

## Declaration of Generative AI and AI-assisted technologies in the writing process

During the preparation of this work CT used Claude AI and ChatGPT to assist with R code development, debugging, and documentation in the statistical analysis process. All code was critically reviewed, edited and approved by CT. During the preparation of this manuscript CT used Microsoft Copilot (institutional license) to help organize and structure ideas and improve the readability of the manuscript. All content has been critically reviewed, edited and approved by all authors. No personal or sensitive data was entered into any AI tool. All authors take full responsibility for the academic integrity and the content of the published article.

## Funding

This publication presents independent research funded by the 10.13039/501100005416Research Council of Norway (Grant reference number: 309264). Haukeland University Hospital is the project owner. The views expressed are those of the author(s) and not necessarily those of the Research Council of Norway or Haukeland University Hospital. This study was undertaken as part of the main author's PhD.

## Declaration of competing interest

BB has developed and is the managing director of the digital treatment program but derives no financial benefits from it. The other authors have no competing interests to declare.

## Data Availability

De-identified data and statistical code can be provided from the corresponding author upon a reasonable request.

## Appendix A Supplementary data

Supplementary file 1 CONSORT_2025_checklist_revised

Supplementary file 2 Study-specific baseline questionnaire_V2

Supplementary file 3 Predictors of dropout

Supplementary file 4 Adherence after 6 months

Supplementary file 5 Mean change by response category 3 m

Supplementary file 6 Meaningful clinical change 6 m

Supplementary file 7 LMM results

Supplementary file 8 CSQ-8 results

## References

[bb0005] Amsallem F., Sanchez S., Armoiry X., Mion F. (2021). Effectiveness of non-pharmacological interventions for irritable bowel syndrome: a systematic review. Evid. Based Complement. Alternat. Med..

[bb0010] Axelsson E., Kern D., Hedman-Lagerlof E., Lindfors P., Palmgren J., Hesser H. (2023). Psychological treatments for irritable bowel syndrome: a comprehensive systematic review and meta-analysis. Cogn. Behav. Ther..

[bb0015] Bellg A.J., Borrelli B., Resnick B., Hecht J., Minicucci D.S., Ory M. (2004). Enhancing treatment fidelity in health behavior change studies: best practices and recommendations from the NIH behavior change consortium. Health Psychol..

[bb0020] Berentsen B., Thuen C., Hillestad E.M.R., Steinsvik E.K., Hausken T., Hatlebakk J.G. (2025). The effects of digital eHealth versus onsite 2-day group-based education in 255 patients with irritable bowel syndrome: cohort study. JMIR Hum. Factors.

[bb0025] Biesiekierski J.R., Manning L.P., Murray H.B., Vlaeyen J.W.S., Ljotsson B., Van Oudenhove L. (2022). Review article: exclude or expose? The paradox of conceptually opposite treatments for irritable bowel syndrome. Aliment. Pharmacol. Ther..

[bb0030] Black C.J., Thakur E.R., Houghton L.A., Quigley E.M.M., Moayyedi P., Ford A.C. (2020). Efficacy of psychological therapies for irritable bowel syndrome: systematic review and network meta-analysis. Gut.

[bb0035] Bosman M., Elsenbruch S., Corsetti M., Tack J., Simren M., Winkens B. (2021). The placebo response rate in pharmacological trials in patients with irritable bowel syndrome: a systematic review and meta-analysis. Lancet Gastroenterol. Hepatol..

[bb0040] Boyce P.M., Talley N.J., Balaam B., Koloski N.A., Truman G. (2003). A randomized controlled trial of cognitive behavior therapy, relaxation training, and routine clinical care for the irritable bowel syndrome. Am. J. Gastroenterol..

[bb0045] Bryan W.H., Amanda R.M., Matthew J.C. (2015). Treatment burden and treatment fatigue as barriers to health. Curr. Opin. Psychol..

[bb0050] Camilleri M. (2021). Diagnosis and treatment of irritable bowel syndrome: a review. Jama.

[bb0055] Carbone F., Van den Houte K., Besard L., Tack C., Arts J., Caenepeel P. (2022). Diet or medication in primary care patients with IBS: the DOMINO study - a randomised trial supported by the Belgian health care knowledge Centre (KCE trials Programme) and the Rome foundation research institute. Gut.

[bb0060] Chey W.D., Keefer L., Whelan K., Gibson P.R. (2021). Behavioral and diet therapies in integrated Care for Patients with Irritable Bowel Syndrome. Gastroenterology.

[bb0065] Christensen H., Mackinnon A. (2006). The law of attrition revisited. J. Med. Internet Res..

[bb0070] Cuijpers P., Reijnders M., Huibers M.J.H. (2019). The role of common factors in psychotherapy outcomes. Annu. Rev. Clin. Psychol..

[bb0075] Devilly G.J., Borkovec T.D. (2000). Psychometric properties of the credibility/expectancy questionnaire. J. Behav. Ther. Exp. Psychiatry.

[bb0080] Dimidi E., McArthur A.J., White R., Whelan K., Lomer M.C.E. (2023). Optimizing educational methods for the low FODMAP diet in disorders of gut-brain interaction: a feasibility randomized controlled trial. Neurogastroenterol. Motil..

[bb0085] Drossman D., Morris C.B., Hu Y., Toner B.B., Diamant N., Whitehead W.E. (2007). Characterization of health related quality of life (HRQOL) for patients with functional bowel disorder (FBD) and its response to treatment. Am. J. Gastroenterol..

[bb0090] Drossman D.A., Patrick D.L., Whitehead W.E., Toner B.B., Diamant N.E., Hu Y. (2000). Further validation of the IBS-QOL: a disease-specific quality-of-life questionnaire. Am. J. Gastroenterol..

[bb0095] D’Silva A., Hua N., Modayil M.V., Seidel J., Marshall D.A. (2025). Digital health interventions are effective for irritable bowel syndrome self-management: a systematic review. Dig. Dis. Sci..

[bb0100] El-Salhy M., Johansson M., Klevstul M., Hatlebakk J.G. (2025). Quality of life, functional impairment and healthcare experiences of patients with irritable bowel syndrome in Norway: an online survey. BMC Gastroenterol..

[bb0105] Flacco M.E., Manzoli L., De Giorgio R., Gasbarrini A., Cicchetti A., Bravi F. (2019). Costs of irritable bowel syndrome in European countries with universal healthcare coverage: a meta-analysis. Eur. Rev. Med. Pharmacol. Sci..

[bb0110] Ford A.C., Lacy B.E., Talley N.J. (2017). Irritable bowel syndrome. N. Engl. J. Med..

[bb0115] Ford A.C., Sperber A.D., Corsetti M., Camilleri M. (2020). Irritable bowel syndrome. Lancet.

[bb0120] Francis C.Y., Morris J., Whorwell P.J. (1997). The irritable bowel severity scoring system: a simple method of monitoring irritable bowel syndrome and its progress. Aliment. Pharmacol. Ther..

[bb0125] Harris P.A., Taylor R., Thielke R., Payne J., Gonzalez N., Conde J.G. (2009). Research electronic data capture (REDCap)--a metadata-driven methodology and workflow process for providing translational research informatics support. J. Biomed. Inform..

[bb0130] Harris P.A., Taylor R., Minor B.L., Elliott V., Fernandez M., O’Neal L. (2019). The REDCap consortium: building an international community of software platform partners. J. Biomed. Inform..

[bb0135] Harvey J.M., Sibelli A., Chalder T., Everitt H., Moss-Morris R., Bishop F.L. (2018). Desperately seeking a cure: treatment seeking and appraisal in irritable bowel syndrome. Br. J. Health Psychol..

[bb0140] Heidelbaugh J.J., Hungin A.P., Palsson O.S., Anastasiou F., Agreus L., Fracasso P. (2025). Perceptions and practices of primary care providers in Europe and the US in the diagnosis and treatment of irritable bowel syndrome: a multinational survey. Neurogastroenterol. Motil..

[bb0145] Hopewell S., Chan A.W., Collins G.S., Hrobjartsson A., Moher D., Schulz K.F. (2025). CONSORT 2025 statement: updated guideline for reporting randomised trials. BMJ.

[bb0150] Jardine J., Nadal C., Robinson S., Enrique A., Hanratty M., Doherty G. (2024). Between rhetoric and reality: real-world barriers to uptake and early engagement in digital mental health interventions. ACM Trans Comput-Hum Interact..

[bb0155] Kaptchuk T.J., Kelley J.M., Conboy L.A., Davis R.B., Kerr C.E., Jacobson E.E. (2008). Components of placebo effect: randomised controlled trial in patients with irritable bowel syndrome. BMJ.

[bb0160] Lacy B.E., Pimentel M., Brenner D.M., Chey W.D., Keefer L.A., Long M.D. (2021). ACG clinical guideline: Management of Irritable Bowel Syndrome. Am. J. Gastroenterol..

[bb0165] Lagun D., Hsieh C.-H., Webster D., Navalpakkam V. (2014). Proceedings of the 37th international ACM SIGIR conference on Research & development in information retrieval.

[bb0170] Laird K.T., Tanner-Smith E.E., Russell A.C., Hollon S.D., Walker L.S. (2016). Short-term and Long-term efficacy of psychological therapies for irritable bowel syndrome: a systematic review and Meta-analysis. Clin. Gastroenterol. Hepatol..

[bb0175] Laird K.T., Tanner-Smith E.E., Russell A.C., Hollon S.D., Walker L.S. (2017). Comparative efficacy of psychological therapies for improving mental health and daily functioning in irritable bowel syndrome: a systematic review and meta-analysis. Clin. Psychol. Rev..

[bb0180] Larsen D.L., Attkisson C.C., Hargreaves W.A., Nguyen T.D. (1979). Assessment of client/patient satisfaction: development of a general scale. Eval. Program Plann..

[bb0185] Ljotsson B., Hedman E., Andersson E., Hesser H., Lindfors P., Hursti T. (2011). Internet-delivered exposure-based treatment vs. stress management for irritable bowel syndrome: a randomized trial. Am. J. Gastroenterol..

[bb0190] Ljotsson B., Hesser H., Andersson E., Lackner J.M., El Alaoui S., Falk L. (2014). Provoking symptoms to relieve symptoms: a randomized controlled dismantling study of exposure therapy in irritable bowel syndrome. Behav. Res. Ther..

[bb0195] Martinez M. (2003). High attrition rates in e-learning: challenges, predictors, and solutions. The eLearning Developers Journal..

[bb0200] McKenzie Y.A., Bowyer R.K., Leach H., Gulia P., Horobin J., O’Sullivan N.A. (2016). British dietetic association systematic review and evidence-based practice guidelines for the dietary management of irritable bowel syndrome in adults (2016 update). J. Hum. Nutr. Diet..

[bb0205] Mearin F., Baró E., Roset M., Badía X., Zárate N., Pérez I. (2004). Clinical patterns over time in irritable bowel syndrome: symptom instability and severity variability. Am. J. Gastroenterol..

[bb0210] Mearin F., Lacy B.E., Chang L., Chey W.D., Lembo A.J., Simren M. (2016). Bowel disorders. Gastroenterology.

[bb0215] Nomeikaite A., Gelezelyte O., Berger T., Andersson G., Kazlauskas E. (2023). Exploring reasons for usage discontinuation in an internet-delivered stress recovery intervention: a qualitative study. Internet Interv..

[bb0220] Oka P., Parr H., Barberio B., Black C.J., Savarino E.V., Ford A.C. (2020). Global prevalence of irritable bowel syndrome according to Rome III or IV criteria: a systematic review and meta-analysis. Lancet Gastroenterol. Hepatol..

[bb0225] Palsson O.S., Whitehead W.E., van Tilburg M.A., Chang L., Chey W., Crowell M.D. (2016). Development and validation of the Rome IV diagnostic questionnaire for adults. Gastroenterology.

[bb0230] Palsson O.S., Whitehead W.E., van Tilburg M.A.L., Chang L., Chey W., Crowell M.D. (2016). Development and validation of the Rome IV diagnostic questionnaire for adults. Gastroenterology.

[bb0235] Pathipati M.P., Scott L.L., Griser A.C., Staller K. (2024). Real-world outcomes for a digital prescription mobile application for adults with irritable bowel syndrome. Neurogastroenterol. Motil..

[bb0240] Nielsen J., Pernice K. (2010).

[bb0245] Ponten M., Jonsjo M., Vadenmark V., Moberg E., Grannas D., Andersson G. (2024). Association between expectations and clinical outcomes in online v. face-to-face therapy - an individual participant data meta-analysis. Psychol. Med..

[bb0250] Ritterband L.M., Thorndike F.P., Cox D.J., Kovatchev B.P., Gonder-Frederick L.A. (2009). A behavior change model for internet interventions. Ann. Behav. Med..

[bb0255] Short C., Rebar A., Plotnikoff R., Vandelanotte C. (2015). Designing engaging online behaviour change interventions: a proposed model of user engagement. Eur. Psychol..

[bb0260] Sieverink F., Kelders S.M., van Gemert-Pijnen J.E. (2017). Clarifying the concept of adherence to eHealth technology: systematic review on when usage becomes adherence. J. Med. Internet Res..

[bb0265] Smith K.A., Ward T., Lambe S., Ostinelli E.G., Blease C., Gant T. (2025). Engagement and attrition in digital mental health: current challenges and potential solutions. Npj. Digit. Med..

[bb0270] Snijkers J.T.W., van den Oever W., Weerts Z., Vork L., Mujagic Z., Leue C. (2021). Examining the optimal cutoff values of HADS, PHQ-9 and GAD-7 as screening instruments for depression and anxiety in irritable bowel syndrome. Neurogastroenterol. Motil..

[bb0275] Tayama J., Hamaguchi T., Koizumi K., Yamamura R., Okubo R., Kawahara J.I. (2024). Efficacy of an eHealth self-management program in reducing irritable bowel syndrome symptom severity: a randomized controlled trial. Sci. Rep..

[bb0280] Thuen C., Hinderaker H.K., Steinsvik E.K., Vikoren L.A.S., Lied G.A., Berentsen B. (2025). Implementing a digital treatment program for patients with irritable bowel syndrome into routine care: a qualitative evaluation of barriers and facilitators perceived by key stakeholders. BMC Health Serv. Res..

[bb0285] Tornkvist N.T., Aziz I., Whitehead W.E., Sperber A.D., Palsson O.S., Hreinsson J.P. (2021). Health care utilization of individuals with Rome IV irritable bowel syndrome in the general population. United European Gastroenterol J.

[bb0290] Tuck C.J., Reed D.E., Muir J.G., Vanner S.J. (2020). Implementation of the low FODMAP diet in functional gastrointestinal symptoms: a real-world experience. Neurogastroenterol. Motil..

[bb0295] Vasant D.H., Paine P.A., Black C.J., Houghton L.A., Everitt H.A., Corsetti M. (2021). British Society of Gastroenterology guidelines on the management of irritable bowel syndrome. Gut.

[bb0300] Wallen H., Ljotsson B., Lindfors P., Forsell E., Hesser H., Svanborg C. (2025). Internet-delivered exposure-based cognitive behavior therapy for irritable bowel syndrome: a clinical effectiveness study. Am. J. Gastroenterol..

[bb0305] Whelan K., Martin L.D., Staudacher H.M., Lomer M.C.E. (2018). The low FODMAP diet in the management of irritable bowel syndrome: an evidence-based review of FODMAP restriction, reintroduction and personalisation in clinical practice. J. Hum. Nutr. Diet..

[bb0310] Yadav Y.S., Eslick G.D., Talley N.J. (2021). Review article: irritable bowel syndrome: natural history, bowel habit stability and overlap with other gastrointestinal disorders. Aliment. Pharmacol. Ther..

[bb0315] Zigmond A.S., Snaith R.P. (1983). The hospital anxiety and depression scale. Acta Psychiatr. Scand..

