# Supplementary material for: Internet-delivered cognitive behavioral therapy and FODMAP diet for adults with irritable bowel syndrome: A four-arm randomized controlled trial
Source: Internet Interv. 2026 Apr 26;44:100949. doi: 10.1016/j.invent.2026.100949 (PMC13141039; doi:10.1016/j.invent.2026.100949)
Supplement: Supplementary file 2 — Study-specific baseline questionnaire_V2 [file mmc2.docx]

Supplementary file 2 Study-specific baseline questionnaire

Dear participant,
Thank you very much for answering these questions. Your responses are anonymized and will help NKFM evaluate the quality of healthcare services at Haukeland University Hospital.

1. **What is your age?**
2. **Gender**
   - Male
   - Female
3. **Weight** [ ]
4. **Height** [ ]
5. **What is your highest level of education?**
   - Primary school level
   - Secondary school level
   - University or college level
6. **What is your employment status?**
   - Student
   - Full-time employment
   - Part-time employment
   - Unemployed (including disability, sick leave)
   - Retired
7. **What is your marital status?**
   - Married
   - Cohabiting
   - Single
8. **How many gastrointestinal examinations have you undergone in total so far?**
   - Gastroscopy [ ]
   - Colonoscopy [ ]
9. **Do you experience any of the following symptoms?**
   - Muscle pain (Fibromyalgia)
   - Headaches
   - Genital discomfort
   - Chronic fatigue
   - Depression
   - Sleep problems
10. **How long have you been living with abdominal complaints?**

- Number of years [ ]

1. **Have you previously tried the low FODMAP diet?**

- Yes
- No

1. **Are you currently following the low FODMAP diet?**

- Yes
- No

1. **To what extent have you experienced symptom relief from the low FODMAP diet?** *(This question appears if you answered “Yes” to question 11 and/or 12)*

- Not at all
- To a small extent
- To a moderate extent
- To a great extent
- To a very great extent
