# Supplementary material for: Internet-delivered cognitive behavioral therapy and FODMAP diet for adults with irritable bowel syndrome: A four-arm randomized controlled trial
Source: Internet Interv. 2026 Apr 26;44:100949. doi: 10.1016/j.invent.2026.100949 (PMC13141039; doi:10.1016/j.invent.2026.100949)
Supplement: Supplementary file 3 — Predictors of dropout [file mmc3.pdf]

Supplementary file 3 Predictors of dropout at 3 months

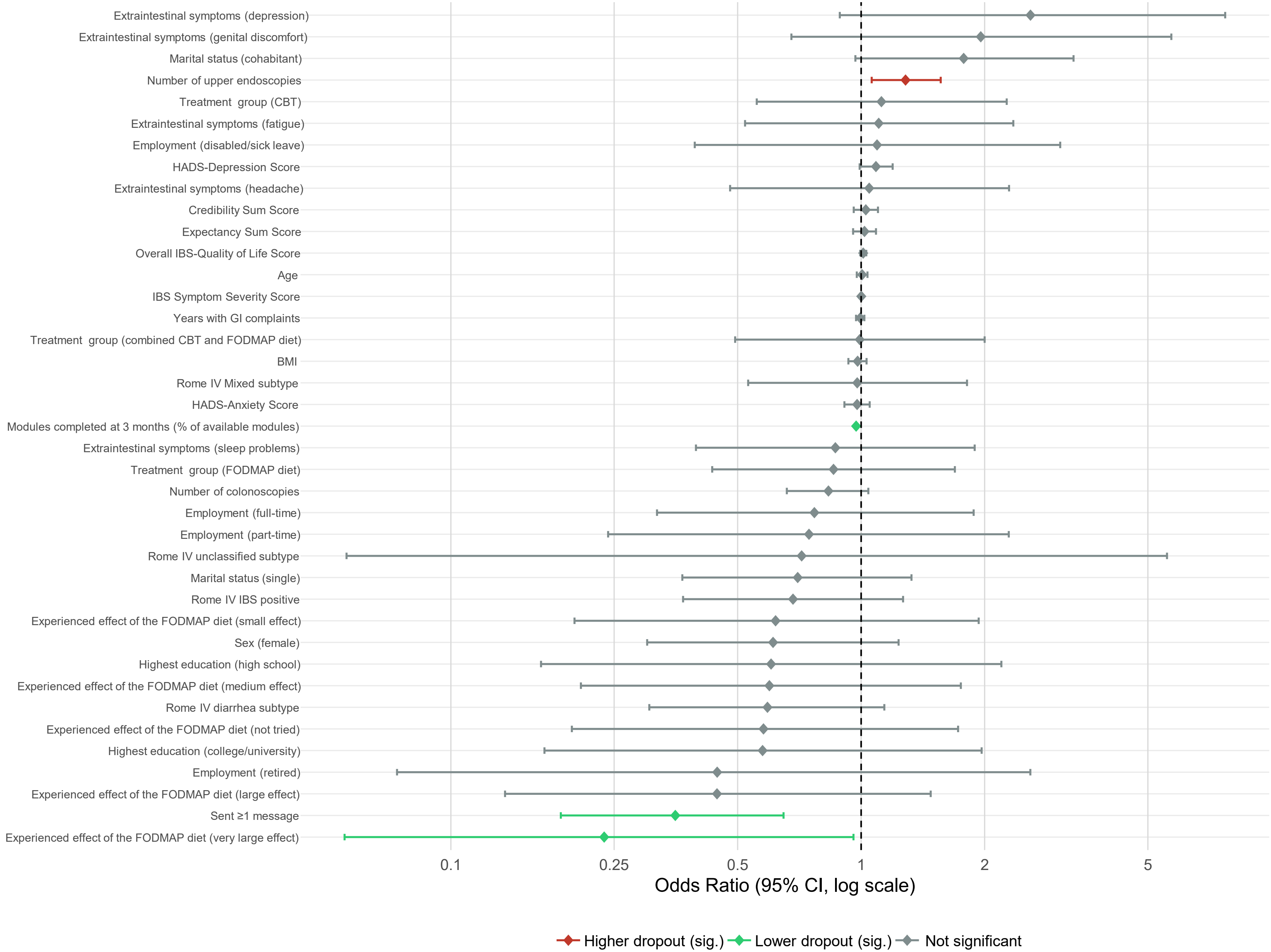

Note: CBT = Cognitive behavioral therapy; FODMAP = fermentable oligosaccharides, disaccharides, monosaccharides, and polyols; GI = gastrointestinal; IBS = irritable bowel syndrome; HADS = Hospital Anxiety and Depression Scale

Supplementary file 3 Predictors of dropout at 6 months

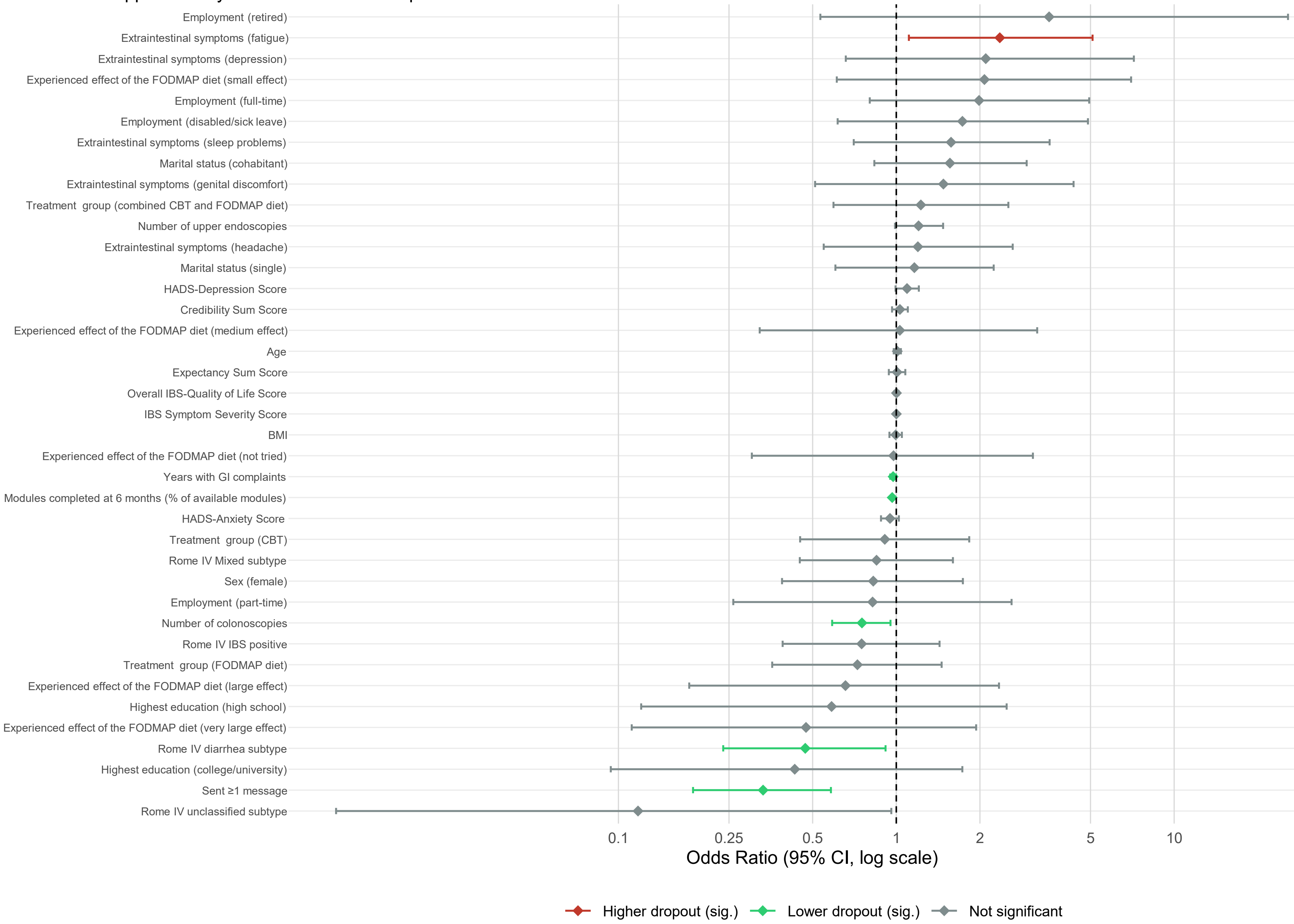

Note: CBT = Cognitive behavioral therapy; FODMAP = fermentable oligosaccharides, disaccharides, monosaccharides, and polyols; GI = gastrointestinal; IBS = irritable bowel syndrome; HADS = Hospital Anxiety and Depression Scale
