# Supplementary material for: Internet-delivered cognitive behavioral therapy and FODMAP diet for adults with irritable bowel syndrome: A four-arm randomized controlled trial
Source: Internet Interv. 2026 Apr 26;44:100949. doi: 10.1016/j.invent.2026.100949 (PMC13141039; doi:10.1016/j.invent.2026.100949)
Supplement: Supplementary file 4 — Adherence after 6 months [file mmc4.docx]

Supplementary file 4 Completion of the first three modules and the intervention modules per group (6 months)

| Group | N | Gastroenterology N (%) | Physiotherapy N (%) | General lifestyle  N (%) | Completion of all three modules  N (%) |
| --- | --- | --- | --- | --- | --- |
| General patient education | 131 | 81 (61.8%) | 79 (60.3%) | 71 (54.2%) | 62 (47.3%) |
| CBT | 145 | 100 (69.0%) | 87 (60.0%) | 79 (54.5%) | 73 (50.3%) |
| FODMAP diet | 139 | 81 (58.3%) | 69 (49.6%) | 63 (45.3%) | 57 (41.0%) |
| Combined CBT and FODMAP diet | 142 | 84 (59.2%) | 76 (53.5%) | 65 (45.8%) | 60 (42.3%) |

| Group | N | CBT  N (%) | FODMAP diet  N (%) | Both CBT and FODMAP |
| --- | --- | --- | --- | --- |
| General patient education | 131 | NA | NA | NA |
| CBT | 145 | 54 (37.2%) | NA | NA |
| FODMAP diet | 139 | NA | 39 (28.1%) | NA |
| Combined CBT and FODMAP diet | 142 | 45 (31.7%) | 31 (21.8%) | 25 (17.6%) |

Note: Adherence is based on logged data and includes all participants who accessed their assigned intervention. Adherence to a module was considered if the participant had navigated through all pages of a module and spent at least 10 min on it. NA indicate that the module was not available for that group.
