# Supplementary material for: Internet-delivered cognitive behavioral therapy and FODMAP diet for adults with irritable bowel syndrome: A four-arm randomized controlled trial
Source: Internet Interv. 2026 Apr 26;44:100949. doi: 10.1016/j.invent.2026.100949 (PMC13141039; doi:10.1016/j.invent.2026.100949)
Supplement: Supplementary file 5 — Mean change by response category 3 m [file mmc5.docx]

Supplementary file 5

Table 1: Mean Change by Response Category at 3 Months

| Outcome | Response Category | Treatment Group | N | Mean Baseline | Mean 3 months | Mean Change (95% CI); % Change (95% CI) |
| --- | --- | --- | --- | --- | --- | --- |
| IBS-SSS | Clinically improved | All groups combined | 164 | 316.5 | 202.5 | -113.9 (95% CI: -122.7 to -105.2); -36.0% (95% CI: -38.8 to -33.2) |
| IBS-SSS | Clinically improved | General patient education | 41 | 315.1 | 200.7 | -114.4 (95% CI: -136.0 to -92.7); -36.3% (95% CI: -43.2 to -29.4) |
| IBS-SSS | Clinically improved | CBT | 42 | 309.0 | 201.2 | -107.8 (95% CI: -124.0 to -91.7); -34.9% (95% CI: -40.1 to -29.7) |
| IBS-SSS | Clinically improved | FODMAP diet | 40 | 326.2 | 203.4 | -122.8 (95% CI: -139.8 to -105.7); -37.6% (95% CI: -42.9 to -32.4) |
| IBS-SSS | Clinically improved | Combined CBT and FODMAP diet | 41 | 316.0 | 204.8 | -111.2 (95% CI: -126.1 to -96.3); -35.2% (95% CI: -39.9 to -30.5) |
| IBS-SSS | No clinical change | All groups combined | 157 | 283.6 | 281.3 | -2.3 (95% CI: -6.8 to 2.1); -0.8% (95% CI: -2.4 to 0.8) |
| IBS-SSS | No clinical change | General patient education | 40 | 281.4 | 282.2 | 0.8 (95% CI: -7.6 to 9.1); 0.3% (95% CI: -2.7 to 3.2) |
| IBS-SSS | No clinical change | CBT | 39 | 297.9 | 297.4 | -0.5 (95% CI: -10.2 to 9.2); -0.2% (95% CI: -3.4 to 3.1) |
| IBS-SSS | No clinical change | FODMAP diet | 38 | 276.4 | 272.6 | -3.8 (95% CI: -13.4 to 5.8); -1.4% (95% CI: -4.9 to 2.1) |
| IBS-SSS | No clinical change | Combined CBT and FODMAP diet | 40 | 278.8 | 273.1 | -5.7 (95% CI: -13.8 to 2.3); -2.1% (95% CI: -4.9 to 0.8) |
| IBS-SSS | Clinically deteriorated | All groups combined | 52 | 261.5 | 344.2 | 82.7 (95% CI: 74.6 to 90.9); 31.6% (95% CI: 28.5 to 34.8) |
| IBS-SSS | Clinically deteriorated | General patient education | 10 | 267.4 | 345.8 | 78.4 (95% CI: 58.5 to 98.3); 29.3% (95% CI: 21.9 to 36.8) |
| IBS-SSS | Clinically deteriorated | CBT | 14 | 272.1 | 346.7 | 74.6 (95% CI: 65.7 to 83.4); 27.4% (95% CI: 24.1 to 30.7) |
| IBS-SSS | Clinically deteriorated | FODMAP diet | 16 | 254.2 | 336.6 | 82.3 (95% CI: 68.0 to 96.6); 32.4% (95% CI: 26.8 to 38.0) |
| IBS-SSS | Clinically deteriorated | Combined CBT and FODMAP diet | 12 | 253.7 | 350.1 | 96.4 (95% CI: 74.5 to 118.4); 38.0% (95% CI: 29.4 to 46.7) |
| Body Image | Clinically improved | All groups combined | 81 | 37.7 | 64.1 | 26.4 (95% CI: 24.2 to 28.7); 70.0% (95% CI: 64.0 to 76.0) |
| Body Image | Clinically improved | General patient education | 17 | 28.3 | 55.5 | 27.2 (95% CI: 22.2 to 32.2); 96.1% (95% CI: 78.3 to 113.9) |
| Body Image | Clinically improved | CBT | 18 | 34.7 | 58.0 | 23.3 (95% CI: 20.5 to 26.0); 67.0% (95% CI: 59.0 to 75.0) |
| Body Image | Clinically improved | FODMAP diet | 24 | 42.7 | 69.1 | 26.4 (95% CI: 22.8 to 29.9); 61.8% (95% CI: 53.5 to 70.1) |
| Body Image | Clinically improved | Combined CBT and FODMAP diet | 22 | 42.0 | 70.5 | 28.4 (95% CI: 22.6 to 34.3); 67.6% (95% CI: 53.7 to 81.5) |
| Body Image | No clinical change | All groups combined | 265 | 44.3 | 45.8 | 1.5 (95% CI: 0.5 to 2.4); 3.3% (95% CI: 1.1 to 5.5) |
| Body Image | No clinical change | General patient education | 66 | 48.3 | 49.8 | 1.5 (95% CI: -0.2 to 3.3); 3.1% (95% CI: -0.5 to 6.7) |
| Body Image | No clinical change | CBT | 72 | 43.6 | 45.1 | 1.5 (95% CI: -0.6 to 3.6); 3.4% (95% CI: -1.4 to 8.2) |
| Body Image | No clinical change | FODMAP diet | 64 | 42.9 | 44.3 | 1.5 (95% CI: -0.4 to 3.4); 3.4% (95% CI: -1.0 to 7.8) |
| Body Image | No clinical change | Combined CBT and FODMAP diet | 63 | 42.4 | 43.8 | 1.4 (95% CI: -0.5 to 3.3); 3.3% (95% CI: -1.2 to 7.8) |
| Body Image | Clinically deteriorated | All groups combined | 26 | 59.4 | 35.3 | -24.0 (95% CI: -27.5 to -20.6); -40.5% (95% CI: -46.3 to -34.7) |
| Body Image | Clinically deteriorated | General patient education | 7 | 72.3 | 37.5 | -34.8 (95% CI: -42.8 to -26.9); -48.1% (95% CI: -59.1 to -37.1) |
| Body Image | Clinically deteriorated | CBT | 5 | 58.8 | 37.5 | -21.2 (95% CI: -26.1 to -16.4); -36.2% (95% CI: -44.5 to -27.8) |
| Body Image | Clinically deteriorated | FODMAP diet | 5 | 53.8 | 32.5 | -21.2 (95% CI: -24.3 to -18.2); -39.5% (95% CI: -45.1 to -34.0) |
| Body Image | Clinically deteriorated | Combined CBT and FODMAP diet | 9 | 52.8 | 34.0 | -18.8 (95% CI: -18.8 to -18.8); -35.5% (95% CI: -35.5 to -35.5) |
| Dysphoria | Clinically improved | All groups combined | 124 | 35.4 | 62.9 | 27.5 (95% CI: 25.3 to 29.8); 77.9% (95% CI: 71.6 to 84.1) |
| Dysphoria | Clinically improved | General patient education | 26 | 31.6 | 61.3 | 29.7 (95% CI: 25.4 to 33.9); 93.9% (95% CI: 80.5 to 107.4) |
| Dysphoria | Clinically improved | CBT | 30 | 34.0 | 58.8 | 24.8 (95% CI: 21.5 to 28.1); 73.0% (95% CI: 63.3 to 82.8) |
| Dysphoria | Clinically improved | FODMAP diet | 31 | 41.8 | 66.0 | 24.2 (95% CI: 20.9 to 27.5); 57.8% (95% CI: 50.0 to 65.7) |
| Dysphoria | Clinically improved | Combined CBT and FODMAP diet | 37 | 33.8 | 64.9 | 31.1 (95% CI: 25.7 to 36.5); 92.0% (95% CI: 76.0 to 108.0) |
| Dysphoria | No clinical change | All groups combined | 222 | 51.6 | 53.7 | 2.0 (95% CI: 1.1 to 3.0); 4.0% (95% CI: 2.2 to 5.7) |
| Dysphoria | No clinical change | General patient education | 55 | 55.1 | 56.8 | 1.7 (95% CI: -0.2 to 3.6); 3.1% (95% CI: -0.4 to 6.6) |
| Dysphoria | No clinical change | CBT | 61 | 48.2 | 51.9 | 3.7 (95% CI: 2.1 to 5.4); 7.8% (95% CI: 4.3 to 11.2) |
| Dysphoria | No clinical change | FODMAP diet | 55 | 50.0 | 51.4 | 1.4 (95% CI: -0.4 to 3.3); 2.8% (95% CI: -0.9 to 6.6) |
| Dysphoria | No clinical change | Combined CBT and FODMAP diet | 51 | 53.8 | 54.8 | 1.0 (95% CI: -0.9 to 3.0); 1.9% (95% CI: -1.7 to 5.5) |
| Dysphoria | Clinically deteriorated | All groups combined | 25 | 61.8 | 37.3 | -24.5 (95% CI: -28.1 to -20.9); -39.7% (95% CI: -45.5 to -33.8) |
| Dysphoria | Clinically deteriorated | General patient education | 9 | 68.1 | 40.3 | -27.8 (95% CI: -36.0 to -19.5); -40.8% (95% CI: -53.0 to -28.7) |
| Dysphoria | Clinically deteriorated | CBT | 4 | 53.1 | 31.2 | -21.9 (95% CI: -27.5 to -16.3); -41.2% (95% CI: -51.7 to -30.7) |
| Dysphoria | Clinically deteriorated | FODMAP diet | 6 | 54.7 | 31.2 | -23.4 (95% CI: -29.3 to -17.6); -42.9% (95% CI: -53.6 to -32.1) |
| Dysphoria | Clinically deteriorated | Combined CBT and FODMAP diet | 6 | 65.1 | 42.7 | -22.4 (95% CI: -28.0 to -16.8); -34.4% (95% CI: -43.0 to -25.8) |
| Food Avoidance | Clinically improved | All groups combined | 104 | 21.2 | 48.2 | 27.0 (95% CI: 24.3 to 29.7); 127.7% (95% CI: 114.8 to 140.5) |
| Food Avoidance | Clinically improved | General patient education | 26 | 20.2 | 45.8 | 25.6 (95% CI: 21.1 to 30.2); 127.0% (95% CI: 104.6 to 149.4) |
| Food Avoidance | Clinically improved | CBT | 23 | 19.2 | 45.7 | 26.4 (95% CI: 22.3 to 30.6); 137.7% (95% CI: 115.9 to 159.6) |
| Food Avoidance | Clinically improved | FODMAP diet | 20 | 20.0 | 44.2 | 24.2 (95% CI: 18.3 to 30.1); 120.8% (95% CI: 91.3 to 150.4) |
| Food Avoidance | Clinically improved | Combined CBT and FODMAP diet | 35 | 23.8 | 53.8 | 30.0 (95% CI: 24.1 to 35.9); 126.0% (95% CI: 101.1 to 150.9) |
| Food Avoidance | No clinical change | All groups combined | 205 | 28.2 | 29.2 | 1.0 (95% CI: 0.1 to 1.8); 3.4% (95% CI: 0.3 to 6.5) |
| Food Avoidance | No clinical change | General patient education | 48 | 31.9 | 33.5 | 1.6 (95% CI: -0.2 to 3.3); 4.9% (95% CI: -0.5 to 10.3) |
| Food Avoidance | No clinical change | CBT | 59 | 25.6 | 25.3 | -0.3 (95% CI: -2.0 to 1.4); -1.1% (95% CI: -7.8 to 5.6) |
| Food Avoidance | No clinical change | FODMAP diet | 54 | 27.8 | 28.6 | 0.8 (95% CI: -0.8 to 2.5); 3.1% (95% CI: -2.9 to 9.0) |
| Food Avoidance | No clinical change | Combined CBT and FODMAP diet | 44 | 28.2 | 30.3 | 2.1 (95% CI: 0.2 to 3.9); 7.4% (95% CI: 0.8 to 13.9) |
| Food Avoidance | Clinically deteriorated | All groups combined | 62 | 49.7 | 25.0 | -24.7 (95% CI: -27.4 to -22.0); -49.7% (95% CI: -55.2 to -44.3) |
| Food Avoidance | Clinically deteriorated | General patient education | 16 | 52.6 | 25.5 | -27.1 (95% CI: -32.6 to -21.6); -51.5% (95% CI: -61.9 to -41.1) |
| Food Avoidance | Clinically deteriorated | CBT | 13 | 46.8 | 27.6 | -19.2 (95% CI: -22.6 to -15.8); -41.1% (95% CI: -48.4 to -33.8) |
| Food Avoidance | Clinically deteriorated | FODMAP diet | 18 | 51.4 | 26.4 | -25.0 (95% CI: -30.4 to -19.6); -48.6% (95% CI: -59.2 to -38.0) |
| Food Avoidance | Clinically deteriorated | Combined CBT and FODMAP diet | 15 | 47.2 | 20.6 | -26.7 (95% CI: -32.7 to -20.7); -56.5% (95% CI: -69.2 to -43.8) |
| Health Worry | Clinically improved | All groups combined | 138 | 40.6 | 67.1 | 26.5 (95% CI: 24.4 to 28.6); 65.3% (95% CI: 60.2 to 70.5) |
| Health Worry | Clinically improved | General patient education | 34 | 35.5 | 61.8 | 26.2 (95% CI: 22.0 to 30.4); 73.8% (95% CI: 62.0 to 85.6) |
| Health Worry | Clinically improved | CBT | 36 | 42.4 | 68.5 | 26.2 (95% CI: 22.1 to 30.2); 61.7% (95% CI: 52.1 to 71.4) |
| Health Worry | Clinically improved | FODMAP diet | 33 | 42.2 | 66.9 | 24.7 (95% CI: 21.0 to 28.5); 58.7% (95% CI: 49.7 to 67.7) |
| Health Worry | Clinically improved | Combined CBT and FODMAP diet | 35 | 42.1 | 71.0 | 28.8 (95% CI: 24.2 to 33.5); 68.4% (95% CI: 57.3 to 79.4) |
| Health Worry | No clinical change | All groups combined | 182 | 57.0 | 57.3 | 0.4 (95% CI: -0.5 to 1.3); 0.6% (95% CI: -0.9 to 2.2) |
| Health Worry | No clinical change | General patient education | 46 | 61.8 | 60.7 | -1.1 (95% CI: -2.7 to 0.6); -1.8% (95% CI: -4.4 to 0.9) |
| Health Worry | No clinical change | CBT | 43 | 54.3 | 54.3 | 0.0 (95% CI: -1.9 to 1.9); 0.0% (95% CI: -3.5 to 3.5) |
| Health Worry | No clinical change | FODMAP diet | 47 | 56.4 | 57.4 | 1.1 (95% CI: -0.7 to 2.8); 1.9% (95% CI: -1.2 to 5.0) |
| Health Worry | No clinical change | Combined CBT and FODMAP diet | 46 | 55.3 | 56.7 | 1.4 (95% CI: -0.5 to 3.4); 2.6% (95% CI: -0.9 to 6.1) |
| Health Worry | Clinically deteriorated | All groups combined | 51 | 67.2 | 43.6 | -23.5 (95% CI: -25.8 to -21.3); -35.0% (95% CI: -38.4 to -31.7) |
| Health Worry | Clinically deteriorated | General patient education | 10 | 76.7 | 54.2 | -22.5 (95% CI: -28.0 to -17.0); -29.3% (95% CI: -36.5 to -22.2) |
| Health Worry | Clinically deteriorated | CBT | 16 | 64.1 | 39.6 | -24.5 (95% CI: -28.8 to -20.1); -38.2% (95% CI: -45.0 to -31.4) |
| Health Worry | Clinically deteriorated | FODMAP diet | 12 | 64.6 | 42.4 | -22.2 (95% CI: -25.9 to -18.6); -34.4% (95% CI: -40.1 to -28.7) |
| Health Worry | Clinically deteriorated | Combined CBT and FODMAP diet | 13 | 66.0 | 41.7 | -24.4 (95% CI: -29.1 to -19.7); -36.9% (95% CI: -44.0 to -29.8) |
| Interference with Activity | Clinically improved | All groups combined | 129 | 36.4 | 60.5 | 24.1 (95% CI: 22.0 to 26.1); 66.1% (95% CI: 60.6 to 71.6) |
| Interference with Activity | Clinically improved | General patient education | 29 | 37.7 | 63.7 | 26.0 (95% CI: 22.2 to 29.8); 69.0% (95% CI: 58.9 to 79.0) |
| Interference with Activity | Clinically improved | CBT | 35 | 33.7 | 55.7 | 22.0 (95% CI: 19.2 to 24.9); 65.5% (95% CI: 57.1 to 73.8) |
| Interference with Activity | Clinically improved | FODMAP diet | 29 | 40.0 | 61.2 | 21.2 (95% CI: 17.9 to 24.5); 52.9% (95% CI: 44.6 to 61.2) |
| Interference with Activity | Clinically improved | Combined CBT and FODMAP diet | 36 | 35.1 | 61.9 | 26.8 (95% CI: 21.6 to 32.0); 76.3% (95% CI: 61.5 to 91.1) |
| Interference with Activity | No clinical change | All groups combined | 211 | 47.0 | 48.3 | 1.3 (95% CI: 0.4 to 2.1); 2.7% (95% CI: 0.9 to 4.4) |
| Interference with Activity | No clinical change | General patient education | 55 | 50.7 | 51.1 | 0.4 (95% CI: -1.3 to 2.1); 0.8% (95% CI: -2.6 to 4.1) |
| Interference with Activity | No clinical change | CBT | 54 | 43.7 | 45.9 | 2.2 (95% CI: 0.8 to 3.7); 5.2% (95% CI: 1.8 to 8.6) |
| Interference with Activity | No clinical change | FODMAP diet | 54 | 46.1 | 48.2 | 2.1 (95% CI: 0.5 to 3.8); 4.6% (95% CI: 1.1 to 8.1) |
| Interference with Activity | No clinical change | Combined CBT and FODMAP diet | 48 | 47.5 | 47.7 | 0.1 (95% CI: -1.7 to 2.0); 0.3% (95% CI: -3.6 to 4.2) |
| Interference with Activity | Clinically deteriorated | All groups combined | 32 | 54.5 | 32.1 | -22.3 (95% CI: -25.3 to -19.4); -41.0% (95% CI: -46.4 to -35.6) |
| Interference with Activity | Clinically deteriorated | General patient education | 6 | 66.1 | 36.9 | -29.2 (95% CI: -39.3 to -19.0); -44.1% (95% CI: -59.5 to -28.8) |
| Interference with Activity | Clinically deteriorated | CBT | 6 | 50.0 | 25.6 | -24.4 (95% CI: -28.2 to -20.6); -48.8% (95% CI: -56.4 to -41.2) |
| Interference with Activity | Clinically deteriorated | FODMAP diet | 10 | 58.9 | 37.9 | -21.1 (95% CI: -26.2 to -15.9); -35.8% (95% CI: -44.5 to -27.0) |
| Interference with Activity | Clinically deteriorated | Combined CBT and FODMAP diet | 10 | 45.7 | 27.5 | -18.2 (95% CI: -21.1 to -15.4); -39.8% (95% CI: -46.1 to -33.6) |
| Relationships | Clinically improved | All groups combined | 109 | 46.2 | 71.6 | 25.5 (95% CI: 23.6 to 27.3); 55.1% (95% CI: 51.0 to 59.2) |
| Relationships | Clinically improved | General patient education | 28 | 46.7 | 71.1 | 24.4 (95% CI: 21.6 to 27.2); 52.2% (95% CI: 46.3 to 58.2) |
| Relationships | Clinically improved | CBT | 31 | 45.4 | 69.4 | 23.9 (95% CI: 20.6 to 27.2); 52.7% (95% CI: 45.4 to 59.9) |
| Relationships | Clinically improved | FODMAP diet | 24 | 52.1 | 74.3 | 22.2 (95% CI: 19.7 to 24.8); 42.7% (95% CI: 37.8 to 47.5) |
| Relationships | Clinically improved | Combined CBT and FODMAP diet | 26 | 41.0 | 72.4 | 31.4 (95% CI: 26.2 to 36.6); 76.6% (95% CI: 63.8 to 89.3) |
| Relationships | No clinical change | All groups combined | 209 | 65.5 | 66.1 | 0.6 (95% CI: -0.3 to 1.5); 0.9% (95% CI: -0.4 to 2.3) |
| Relationships | No clinical change | General patient education | 49 | 69.0 | 70.4 | 1.4 (95% CI: -0.4 to 3.1); 2.0% (95% CI: -0.5 to 4.5) |
| Relationships | No clinical change | CBT | 47 | 64.7 | 64.9 | 0.2 (95% CI: -1.8 to 2.1); 0.3% (95% CI: -2.7 to 3.3) |
| Relationships | No clinical change | FODMAP diet | 58 | 65.4 | 65.7 | 0.4 (95% CI: -1.4 to 2.1); 0.6% (95% CI: -2.1 to 3.2) |
| Relationships | No clinical change | Combined CBT and FODMAP diet | 55 | 63.0 | 63.6 | 0.6 (95% CI: -1.2 to 2.4); 1.0% (95% CI: -1.9 to 3.8) |
| Relationships | Clinically deteriorated | All groups combined | 54 | 71.8 | 49.8 | -21.9 (95% CI: -23.7 to -20.1); -30.5% (95% CI: -33.0 to -28.0) |
| Relationships | Clinically deteriorated | General patient education | 13 | 73.1 | 48.7 | -24.4 (95% CI: -29.7 to -19.0); -33.3% (95% CI: -40.7 to -26.0) |
| Relationships | Clinically deteriorated | CBT | 17 | 67.2 | 45.6 | -21.6 (95% CI: -24.0 to -19.1); -32.1% (95% CI: -35.8 to -28.5) |
| Relationships | Clinically deteriorated | FODMAP diet | 11 | 75.0 | 54.5 | -20.5 (95% CI: -23.8 to -17.1); -27.3% (95% CI: -31.8 to -22.8) |
| Relationships | Clinically deteriorated | Combined CBT and FODMAP diet | 13 | 73.7 | 52.6 | -21.2 (95% CI: -24.1 to -18.2); -28.7% (95% CI: -32.8 to -24.6) |
| Social Reaction | Clinically improved | All groups combined | 83 | 39.6 | 66.7 | 27.1 (95% CI: 25.2 to 29.0); 68.4% (95% CI: 63.7 to 73.2) |
| Social Reaction | Clinically improved | General patient education | 19 | 39.5 | 65.1 | 25.7 (95% CI: 22.4 to 28.9); 65.0% (95% CI: 56.8 to 73.2) |
| Social Reaction | Clinically improved | CBT | 20 | 41.9 | 67.8 | 25.9 (95% CI: 22.5 to 29.4); 61.9% (95% CI: 53.6 to 70.2) |
| Social Reaction | Clinically improved | FODMAP diet | 26 | 39.4 | 64.9 | 25.5 (95% CI: 22.7 to 28.3); 64.6% (95% CI: 57.6 to 71.7) |
| Social Reaction | Clinically improved | Combined CBT and FODMAP diet | 18 | 37.5 | 69.8 | 32.3 (95% CI: 26.9 to 37.7); 86.1% (95% CI: 71.8 to 100.4) |
| Social Reaction | No clinical change | All groups combined | 245 | 59.8 | 59.8 | -0.0 (95% CI: -1.0 to 1.0); -0.0% (95% CI: -1.7 to 1.6) |
| Social Reaction | No clinical change | General patient education | 61 | 63.0 | 63.1 | 0.1 (95% CI: -1.9 to 2.1); 0.2% (95% CI: -3.1 to 3.4) |
| Social Reaction | No clinical change | CBT | 59 | 56.2 | 57.1 | 0.8 (95% CI: -1.3 to 3.0); 1.5% (95% CI: -2.3 to 5.4) |
| Social Reaction | No clinical change | FODMAP diet | 59 | 62.7 | 62.3 | -0.4 (95% CI: -2.5 to 1.7); -0.7% (95% CI: -4.0 to 2.7) |
| Social Reaction | No clinical change | Combined CBT and FODMAP diet | 66 | 57.6 | 57.0 | -0.6 (95% CI: -2.3 to 1.1); -1.0% (95% CI: -4.0 to 2.0) |
| Social Reaction | Clinically deteriorated | All groups combined | 44 | 75.1 | 50.9 | -24.3 (95% CI: -26.2 to -22.4); -32.3% (95% CI: -34.8 to -29.8) |
| Social Reaction | Clinically deteriorated | General patient education | 10 | 72.5 | 46.9 | -25.6 (95% CI: -29.9 to -21.4); -35.3% (95% CI: -41.2 to -29.5) |
| Social Reaction | Clinically deteriorated | CBT | 16 | 71.9 | 47.3 | -24.6 (95% CI: -28.4 to -20.8); -34.2% (95% CI: -39.5 to -29.0) |
| Social Reaction | Clinically deteriorated | FODMAP diet | 8 | 75.8 | 53.1 | -22.7 (95% CI: -25.9 to -19.4); -29.9% (95% CI: -34.1 to -25.6) |
| Social Reaction | Clinically deteriorated | Combined CBT and FODMAP diet | 10 | 82.5 | 58.8 | -23.8 (95% CI: -26.8 to -20.7); -28.8% (95% CI: -32.5 to -25.1) |
| Sexual Function | Clinically improved | All groups combined | 82 | 34.8 | 68.6 | 33.8 (95% CI: 31.3 to 36.4); 97.4% (95% CI: 90.1 to 104.7) |
| Sexual Function | Clinically improved | General patient education | 21 | 28.0 | 61.3 | 33.3 (95% CI: 27.6 to 39.0); 119.1% (95% CI: 98.8 to 139.5) |
| Sexual Function | Clinically improved | CBT | 17 | 35.3 | 68.4 | 33.1 (95% CI: 27.6 to 38.6); 93.7% (95% CI: 78.1 to 109.4) |
| Sexual Function | Clinically improved | FODMAP diet | 19 | 42.1 | 79.6 | 37.5 (95% CI: 32.9 to 42.1); 89.1% (95% CI: 78.2 to 100.0) |
| Sexual Function | Clinically improved | Combined CBT and FODMAP diet | 25 | 34.5 | 66.5 | 32.0 (95% CI: 27.5 to 36.5); 92.8% (95% CI: 79.7 to 105.8) |
| Sexual Function | No clinical change | All groups combined | 252 | 57.3 | 56.6 | -0.6 (95% CI: -1.8 to 0.5); -1.1% (95% CI: -3.1 to 0.8) |
| Sexual Function | No clinical change | General patient education | 58 | 60.8 | 60.8 | 0.0 (95% CI: -2.2 to 2.2); 0.0% (95% CI: -3.6 to 3.6) |
| Sexual Function | No clinical change | CBT | 70 | 54.5 | 52.9 | -1.6 (95% CI: -3.7 to 0.5); -3.0% (95% CI: -6.8 to 0.9) |
| Sexual Function | No clinical change | FODMAP diet | 65 | 56.5 | 56.7 | 0.2 (95% CI: -2.1 to 2.4); 0.3% (95% CI: -3.6 to 4.3) |
| Sexual Function | No clinical change | Combined CBT and FODMAP diet | 59 | 58.1 | 57.0 | -1.1 (95% CI: -3.4 to 1.3); -1.8% (95% CI: -5.9 to 2.3) |
| Sexual Function | Clinically deteriorated | All groups combined | 35 | 72.9 | 39.6 | -33.2 (95% CI: -37.5 to -29.0); -45.6% (95% CI: -51.4 to -39.7) |
| Sexual Function | Clinically deteriorated | General patient education | 10 | 72.5 | 33.8 | -38.8 (95% CI: -48.0 to -29.5); -53.4% (95% CI: -66.2 to -40.7) |
| Sexual Function | Clinically deteriorated | CBT | 8 | 70.3 | 37.5 | -32.8 (95% CI: -42.0 to -23.6); -46.7% (95% CI: -59.7 to -33.6) |
| Sexual Function | Clinically deteriorated | FODMAP diet | 8 | 68.8 | 35.9 | -32.8 (95% CI: -43.1 to -22.5); -47.7% (95% CI: -62.7 to -32.8) |
| Sexual Function | Clinically deteriorated | Combined CBT and FODMAP diet | 9 | 79.2 | 51.4 | -27.8 (95% CI: -31.4 to -24.2); -35.1% (95% CI: -39.6 to -30.5) |
| IBS-QoL Overall Score | Clinically improved | All groups combined | 80 | 38.5 | 62.9 | 24.5 (95% CI: 22.3 to 26.7); 63.6% (95% CI: 57.8 to 69.4) |
| IBS-QoL Overall Score | Clinically improved | General patient education | 17 | 35.0 | 60.3 | 25.4 (95% CI: 21.3 to 29.5); 72.5% (95% CI: 60.8 to 84.2) |
| IBS-QoL Overall Score | Clinically improved | CBT | 20 | 40.4 | 62.6 | 22.2 (95% CI: 18.9 to 25.5); 55.0% (95% CI: 46.7 to 63.2) |
| IBS-QoL Overall Score | Clinically improved | FODMAP diet | 19 | 42.6 | 63.4 | 20.8 (95% CI: 17.8 to 23.9); 48.9% (95% CI: 41.7 to 56.0) |
| IBS-QoL Overall Score | Clinically improved | Combined CBT and FODMAP diet | 24 | 36.1 | 64.7 | 28.6 (95% CI: 23.2 to 34.1); 79.4% (95% CI: 64.4 to 94.5) |
| IBS-QoL Overall Score | No clinical change | All groups combined | 278 | 49.6 | 51.8 | 2.2 (95% CI: 1.4 to 3.1); 4.5% (95% CI: 2.8 to 6.2) |
| IBS-QoL Overall Score | No clinical change | General patient education | 67 | 52.6 | 55.4 | 2.8 (95% CI: 1.1 to 4.5); 5.3% (95% CI: 2.1 to 8.5) |
| IBS-QoL Overall Score | No clinical change | CBT | 72 | 46.3 | 48.6 | 2.3 (95% CI: 0.7 to 3.9); 5.0% (95% CI: 1.6 to 8.4) |
| IBS-QoL Overall Score | No clinical change | FODMAP diet | 70 | 49.5 | 52.4 | 2.8 (95% CI: 1.1 to 4.6); 5.7% (95% CI: 2.2 to 9.3) |
| IBS-QoL Overall Score | No clinical change | Combined CBT and FODMAP diet | 69 | 50.3 | 51.3 | 1.0 (95% CI: -0.8 to 2.8); 2.0% (95% CI: -1.6 to 5.6) |
| IBS-QoL Overall Score | Clinically deteriorated | All groups combined | 14 | 63.1 | 41.4 | -21.6 (95% CI: -25.7 to -17.6); -34.3% (95% CI: -40.8 to -27.8) |
| IBS-QoL Overall Score | Clinically deteriorated | General patient education | 6 | 66.5 | 42.4 | -24.1 (95% CI: -31.7 to -16.6); -36.3% (95% CI: -47.6 to -24.9) |
| IBS-QoL Overall Score | Clinically deteriorated | CBT | 3 | 58.1 | 40.4 | -17.6 (95% CI: -21.0 to -14.3); -30.4% (95% CI: -36.1 to -24.7) |
| IBS-QoL Overall Score | Clinically deteriorated | FODMAP diet | 4 | 57.2 | 39.0 | -18.2 (95% CI: -23.2 to -13.2); -31.8% (95% CI: -40.6 to -23.0) |
| IBS-QoL Overall Score | Clinically deteriorated | Combined CBT and FODMAP diet | 1 | 80.9 | 48.5 | -32.4 (95% CI: NA to NA); -40.0% (95% CI: NA to NA) |

Note: For IBS-SSS: negative values indicate symptom reduction (improvement). For QoL scores: positive values indicate improvement. Improvement for IBS-SSS is 50-point decrease, no clinical change is 49-point decrease to 49-point increase, deterioration is 50-point increase. Improvement for IBS-QoL is 14-point increase, no clinical change is 13-point increase to 13-point decrease, deterioration is 13-point decrease.
