# Supplementary material for: Internet-delivered cognitive behavioral therapy and FODMAP diet for adults with irritable bowel syndrome: A four-arm randomized controlled trial
Source: Internet Interv. 2026 Apr 26;44:100949. doi: 10.1016/j.invent.2026.100949 (PMC13141039; doi:10.1016/j.invent.2026.100949)
Supplement: Supplementary file 6 — Meaningful clinical change 6 m [file mmc6.docx]

Supplementary file 6 Meaningful clinical change after 6 months

| Outcome | Treatment Group | N | Clinically Deteriorated | No clinical Change | Clinically Improved | Between-group p-value clinically improvement | Between-group OR (95% CI) |
| --- | --- | --- | --- | --- | --- | --- | --- |
| **IBS-SSS** | General patient education | 66 | 8 (12.1%) | 28 (42.4%) | 30 (45.5%) | 0.990 | 0.98 [0.50, 1.93] |
|  | CBT | 71 | 8 (11.3%) | 31 (43.7%) | 32 (45.1%) |  |  |
|  | FODMAP diet | 61 | 7 (11.5%) | 27 (44.3%) | 27 (44.3%) |  |  |
|  | Combined CBT and FODMAP diet | 55 | 7 (12.7%) | 22 (40%) | 26 (47.3%) |  |  |
| **Body Image** | General patient education | 66 | 4 (6.1%) | 44 (66.7%) | 18 (27.3%) | 0.865 | 1.30 [0.62, 2.73] |
|  | CBT | 70 | 8 (11.4%) | 39 (55.7%) | 23 (32.9%) |  |  |
|  | FODMAP diet | 60 | 1 (1.7%) | 43 (71.7%) | 16 (26.7%) |  |  |
|  | Combined CBT and FODMAP diet | 55 | 4 (7.3%) | 35 (63.6%) | 16 (29.1%) |  |  |
| **Dysphoria** | General patient education | 66 | 7 (10.6%) | 37 (56.1%) | 22 (33.3%) | 0.313 | 1.59 [0.79, 3.19] |
|  | CBT | 70 | 4 (5.7%) | 35 (50%) | 31 (44.3%) |  |  |
|  | FODMAP diet | 60 | 2 (3.3%) | 37 (61.7%) | 21 (35%) |  |  |
|  | Combined CBT and FODMAP diet | 55 | 3 (5.5%) | 26 (47.3%) | 26 (47.3%) |  |  |
| **Food Avoidance** | General patient education | 66 | 13 (19.7%) | 34 (51.5%) | 19 (28.8%) | 0.501 | 1.65 [0.81, 3.37] |
|  | CBT | 70 | 9 (12.9%) | 33 (47.1%) | 28 (40%) |  |  |
|  | FODMAP diet | 60 | 11 (18.3%) | 29 (48.3%) | 20 (33.3%) |  |  |
|  | Combined CBT and FODMAP diet | 55 | 8 (14.5%) | 31 (56.4%) | 16 (29.1%) |  |  |
| **Health Worry** | General patient education | 66 | 14 (21.2%) | 29 (43.9%) | 23 (34.8%) | 0.385 | 1.40 [0.70, 2.80] |
|  | CBT | 70 | 6 (8.6%) | 34 (48.6%) | 30 (42.9%) |  |  |
|  | FODMAP diet | 60 | 12 (20%) | 26 (43.3%) | 22 (36.7%) |  |  |
|  | Combined CBT and FODMAP diet | 55 | 4 (7.3%) | 24 (43.6%) | 27 (49.1%) |  |  |
| **Interference with Activity** | General patient education | 66 | 6 (9.1%) | 35 (53%) | 25 (37.9%) | 0.448 | 1.38 [0.70, 2.74] |
|  | CBT | 70 | 7 (10%) | 31 (44.3%) | 32 (45.7%) |  |  |
|  | FODMAP diet | 60 | 5 (8.3%) | 34 (56.7%) | 21 (35%) |  |  |
|  | Combined CBT and FODMAP diet | 55 | 2 (3.6%) | 27 (49.1%) | 26 (47.3%) |  |  |
| **Relationships** | General patient education | 66 | 8 (12.1%) | 38 (57.6%) | 20 (30.3%) | 0.765 | 1.44 [0.71, 2.94] |
|  | CBT | 70 | 10 (14.3%) | 33 (47.1%) | 27 (38.6%) |  |  |
|  | FODMAP diet | 60 | 10 (16.7%) | 30 (50%) | 20 (33.3%) |  |  |
|  | Combined CBT and FODMAP diet | 55 | 2 (3.6%) | 33 (60%) | 20 (36.4%) |  |  |
| **Social Reaction** | General patient education | 66 | 8 (12.1%) | 43 (65.2%) | 15 (22.7%) | 0.414 | 1.46 [0.67, 3.15] |
|  | CBT | 70 | 7 (10%) | 42 (60%) | 21 (30%) |  |  |
|  | FODMAP diet | 60 | 6 (10%) | 38 (63.3%) | 16 (26.7%) |  |  |
|  | Combined CBT and FODMAP diet | 55 | 4 (7.3%) | 31 (56.4%) | 20 (36.4%) |  |  |
| **Sexual Function** | General patient education | 65 | 10 (15.4%) | 43 (66.2%) | 12 (18.5%) | 0.281 | 1.31 [0.57, 3.03] |
|  | CBT | 70 | 12 (17.1%) | 42 (60%) | 16 (22.9%) |  |  |
|  | FODMAP diet | 59 | 9 (15.3%) | 33 (55.9%) | 17 (28.8%) |  |  |
|  | Combined CBT and FODMAP diet | 55 | 5 (9.1%) | 32 (58.2%) | 18 (32.7%) |  |  |
| **IBS-QoL Overall Score** | General patient education | 66 | 4 (6.1%) | 47 (71.2%) | 15 (22.7%) | 0.276 | 2.01 [0.95, 4.26] |
|  | CBT | 70 | 4 (5.7%) | 40 (57.1%) | 26 (37.1%) |  |  |
|  | FODMAP diet | 60 | 2 (3.3%) | 41 (68.3%) | 17 (28.3%) |  |  |
|  | Combined CBT and FODMAP diet | 55 | 2 (3.6%) | 34 (61.8%) | 19 (34.5%) |  |  |
| *^1^*Note: Number of participants categorized as Clinically Deteriorated, No clinical Change or Clinically Improved are calculated using cut-offs that are regarded as clinical meaningful and presented as n (%); IBS-SSS threshold = 50 points (lower score indicate improvement); IBS-QoL subscales threshold = 14 points (higher score indicate improvement); Between-group differences after six months were analyzed using chi-square tests with effect sizes reported as Cramer's V (95% CI); IBS-SSS = Irritable Bowel Syndrome Symptom Severity Score; IBS-QoL = Irritable Bowel Syndrome Quality of Life. | | | | | | | |
