# Supplementary material for: Internet-delivered cognitive behavioral therapy and FODMAP diet for adults with irritable bowel syndrome: A four-arm randomized controlled trial
Source: Internet Interv. 2026 Apr 26;44:100949. doi: 10.1016/j.invent.2026.100949 (PMC13141039; doi:10.1016/j.invent.2026.100949)
Supplement: Supplementary file 7 — LMM results [file mmc7.docx]

Supplementary file 7 Results from linear mixed models

|  | | | |
| --- | --- | --- | --- |
|  | | | |
|  | Beta (95% CI) | SE | p-value |
| **IBS-SSS (n = 557)** | | | |
| Intercept (General patient education group) | 300.1 (285.5, 314.8) | 7.5 | <0.001 |
| CBT group | 0.2 (-20.0, 20.4) | 10.3 | 0.98 |
| FODMAP diet group | -9.8 (-30.2, 10.6) | 10.4 | 0.35 |
| Combined CBT and FODMAP diet group | -9.8 (-30.1, 10.5) | 10.4 | 0.34 |
| Time (3 month) | -45.1 (-62.1, -28.2) | 8.6 | <0.001 |
| Time (6 month) | -63.3 (-82.4, -44.2) | 9.7 | <0.001 |
| CBT group * Time (3 month) | 7.6 (-16.0, 31.2) | 12.0 | 0.53 |
| FODMAP diet group * Time (3 month) | 7.1 (-16.6, 30.8) | 12.1 | 0.56 |
| Combined CBT and FODMAP diet group * Time (3 month) | 6.3 (-17.5, 30.0) | 12.1 | 0.60 |
| CBT group * Time (6 month) | 2.3 (-24.2, 28.8) | 13.5 | 0.86 |
| FODMAP diet group * Time (6 month) | 18.3 (-9.0, 45.7) | 14.0 | 0.19 |
| Combined CBT and FODMAP diet group * Time (6 month) | 12.5 (-15.5, 40.4) | 14.3 | 0.38 |
| **Sexual Score (n = 553)** | | | |
| Intercept (General patient education group) | 56.0 (50.9, 61.1) | 2.6 | <0.001 |
| CBT group | -3.3 (-10.4, 3.7) | 3.6 | 0.36 |
| FODMAP diet group | -2.0 (-9.1, 5.1) | 3.6 | 0.59 |
| Combined CBT and FODMAP diet group | 0.5 (-6.6, 7.5) | 3.6 | 0.90 |
| Time (3 month) | 3.0 (-1.5, 7.6) | 2.3 | 0.19 |
| Time (6 month) | 1.6 (-3.5, 6.7) | 2.6 | 0.54 |
| CBT group * Time (3 month) | -1.2 (-7.5, 5.1) | 3.2 | 0.72 |
| FODMAP diet group * Time (3 month) | 2.0 (-4.3, 8.3) | 3.2 | 0.54 |
| Combined CBT and FODMAP diet group * Time (3 month) | 1.4 (-4.9, 7.8) | 3.2 | 0.66 |
| CBT group * Time (6 month) | 3.4 (-3.7, 10.5) | 3.6 | 0.34 |
| FODMAP diet group * Time (6 month) | 4.9 (-2.5, 12.2) | 3.8 | 0.20 |
| Combined CBT and FODMAP diet group * Time (6 month) | 4.7 (-2.8, 12.1) | 3.8 | 0.22 |
| **Social Relation Score (n = 554)** | | | |
| Intercept (General patient education group) | 57.6 (53.6, 61.7) | 2.1 | <0.001 |
| CBT group | -1.5 (-7.1, 4.1) | 2.9 | 0.60 |
| FODMAP diet group | -0.7 (-6.3, 5.0) | 2.9 | 0.82 |
| Combined CBT and FODMAP diet group | 0.6 (-5.1, 6.3) | 2.9 | 0.84 |
| Time (3 month) | 3.0 (-0.5, 6.6) | 1.8 | 0.10 |
| Time (6 month) | 5.9 (1.9, 10.0) | 2.1 | 0.00 |
| CBT group * Time (3 month) | -1.3 (-6.3, 3.7) | 2.5 | 0.62 |
| FODMAP diet group * Time (3 month) | 1.9 (-3.1, 6.9) | 2.6 | 0.46 |
| Combined CBT and FODMAP diet group * Time (3 month) | -0.3 (-5.3, 4.7) | 2.6 | 0.91 |
| CBT group * Time (6 month) | 1.4 (-4.3, 7.0) | 2.9 | 0.63 |
| FODMAP diet group * Time (6 month) | 1.3 (-4.5, 7.1) | 3.0 | 0.66 |
| Combined CBT and FODMAP diet group * Time (6 month) | 2.1 (-3.8, 8.1) | 3.0 | 0.48 |
| **Relationships Score (n = 554)** | | | |
| Intercept (General patient education group) | 62.9 (58.8, 67.1) | 2.1 | <0.001 |
| CBT group | -4.4 (-10.2, 1.3) | 2.9 | 0.13 |
| FODMAP diet group | -0.2 (-6.0, 5.6) | 3.0 | 0.94 |
| Combined CBT and FODMAP diet group | -0.7 (-6.4, 5.1) | 3.0 | 0.82 |
| Time (3 month) | 4.7 (1.2, 8.3) | 1.8 | 0.01 |
| Time (6 month) | 4.9 (1.0, 8.9) | 2.0 | 0.02 |
| CBT group * Time (3 month) | -0.6 (-5.5, 4.3) | 2.5 | 0.80 |
| FODMAP diet group * Time (3 month) | -1.1 (-6.0, 3.8) | 2.5 | 0.66 |
| Combined CBT and FODMAP diet group * Time (3 month) | 0.4 (-4.5, 5.3) | 2.5 | 0.88 |
| CBT group * Time (6 month) | 3.4 (-2.1, 8.9) | 2.8 | 0.23 |
| FODMAP diet group * Time (6 month) | 0.3 (-5.4, 6.0) | 2.9 | 0.92 |
| Combined CBT and FODMAP diet group * Time (6 month) | 1.8 (-4.0, 7.6) | 3.0 | 0.53 |
| **Interference with Activity Score (n = 554)** | | | |
| Intercept (General patient education group) | 47.0 (42.9, 51.1) | 2.1 | <0.001 |
| CBT group | -5.0 (-10.6, 0.7) | 2.9 | 0.09 |
| FODMAP diet group | 0.0 (-5.7, 5.7) | 2.9 | 1.00 |
| Combined CBT and FODMAP diet group | -1.6 (-7.3, 4.1) | 2.9 | 0.58 |
| Time (3 month) | 6.8 (3.5, 10.1) | 1.7 | <0.001 |
| Time (6 month) | 9.3 (5.6, 13.1) | 1.9 | <0.001 |
| CBT group * Time (3 month) | 0.7 (-3.9, 5.3) | 2.3 | 0.77 |
| FODMAP diet group * Time (3 month) | -1.5 (-6.1, 3.1) | 2.4 | 0.52 |
| Combined CBT and FODMAP diet group * Time (3 month) | 1.0 (-3.7, 5.6) | 2.4 | 0.69 |
| CBT group * Time (6 month) | 3.0 (-2.2, 8.1) | 2.6 | 0.26 |
| FODMAP diet group * Time (6 month) | -2.2 (-7.6, 3.1) | 2.7 | 0.41 |
| Combined CBT and FODMAP diet group * Time (6 month) | 2.1 (-3.4, 7.5) | 2.8 | 0.45 |
| **Health Worry Score (n = 554)** | | | |
| Intercept (General patient education group) | 51.8 (47.7, 55.9) | 2.1 | <0.001 |
| CBT group | -0.3 (-6.0, 5.3) | 2.9 | 0.90 |
| FODMAP diet group | -0.8 (-6.4, 4.9) | 2.9 | 0.79 |
| Combined CBT and FODMAP diet group | 1.3 (-4.3, 7.0) | 2.9 | 0.64 |
| Time (3 month) | 7.4 (3.6, 11.3) | 2.0 | <0.001 |
| Time (6 month) | 8.4 (4.1, 12.7) | 2.2 | <0.001 |
| CBT group * Time (3 month) | -1.6 (-7.0, 3.7) | 2.7 | 0.55 |
| FODMAP diet group * Time (3 month) | -0.7 (-6.1, 4.7) | 2.8 | 0.81 |
| Combined CBT and FODMAP diet group * Time (3 month) | 0.2 (-5.1, 5.6) | 2.7 | 0.93 |
| CBT group * Time (6 month) | 3.7 (-2.3, 9.8) | 3.1 | 0.22 |
| FODMAP diet group * Time (6 month) | -3.1 (-9.3, 3.2) | 3.2 | 0.33 |
| Combined CBT and FODMAP diet group * Time (6 month) | 2.0 (-4.3, 8.4) | 3.2 | 0.53 |
| **Food Avoidance Score (n = 554)** | | | |
| Intercept (General patient education group) | 32.1 (27.9, 36.3) | 2.1 | <0.001 |
| CBT group | -2.0 (-7.7, 3.8) | 2.9 | 0.50 |
| FODMAP diet group | -2.5 (-8.3, 3.3) | 3.0 | 0.40 |
| Combined CBT and FODMAP diet group | -3.0 (-8.8, 2.7) | 2.9 | 0.30 |
| Time (3 month) | 3.5 (-0.5, 7.4) | 2.0 | 0.09 |
| Time (6 month) | 7.0 (2.6, 11.4) | 2.3 | 0.00 |
| CBT group * Time (3 month) | -0.9 (-6.4, 4.6) | 2.8 | 0.74 |
| FODMAP diet group * Time (3 month) | -2.3 (-7.8, 3.2) | 2.8 | 0.41 |
| Combined CBT and FODMAP diet group * Time (3 month) | 4.6 (-0.9, 10.1) | 2.8 | 0.10 |
| CBT group * Time (6 month) | 1.6 (-4.5, 7.8) | 3.2 | 0.60 |
| FODMAP diet group * Time (6 month) | -3.2 (-9.5, 3.2) | 3.3 | 0.33 |
| Combined CBT and FODMAP diet group * Time (6 month) | 1.1 (-5.4, 7.6) | 3.3 | 0.74 |
| **Dysphoria Score (n = 554)** | | | |
| Intercept (General patient education group) | 49.6 (45.3, 53.9) | 2.2 | <0.001 |
| CBT group | -5.9 (-11.8, -0.0) | 3.0 | 0.05 |
| FODMAP diet group | -3.1 (-9.1, 2.8) | 3.0 | 0.30 |
| Combined CBT and FODMAP diet group | -0.2 (-6.1, 5.8) | 3.0 | 0.96 |
| Time (3 month) | 6.8 (3.3, 10.4) | 1.8 | <0.001 |
| Time (6 month) | 8.0 (3.9, 12.0) | 2.1 | <0.001 |
| CBT group * Time (3 month) | 2.5 (-2.5, 7.5) | 2.5 | 0.32 |
| FODMAP diet group * Time (3 month) | 0.8 (-4.2, 5.9) | 2.6 | 0.75 |
| Combined CBT and FODMAP diet group * Time (3 month) | 3.8 (-1.2, 8.8) | 2.6 | 0.14 |
| **CBT group * Time (6 month)** | **7.8 (2.1, 13.4)** | **2.9** | **0.01** |
| FODMAP diet group * Time (6 month) | 2.2 (-3.6, 8.0) | 3.0 | 0.46 |
| **Combined CBT and FODMAP diet group * Time (6 month)** | **6.4 (0.4, 12.3)** | **3.0** | **0.04** |
| **Body Image Score (n = 554)** | | | |
| Intercept (General patient education group) | 46.1 (42.1, 50.0) | 2.0 | <0.001 |
| CBT group | -3.3 (-8.8, 2.1) | 2.8 | 0.23 |
| FODMAP diet group | -3.3 (-8.8, 2.1) | 2.8 | 0.23 |
| Combined CBT and FODMAP diet group | -1.6 (-7.0, 3.9) | 2.8 | 0.58 |
| Time (3 month) | 3.6 (0.4, 6.9) | 1.7 | 0.03 |
| Time (6 month) | 7.8 (4.1, 11.5) | 1.9 | <0.001 |
| CBT group * Time (3 month) | 0.8 (-3.8, 5.3) | 2.3 | 0.74 |
| FODMAP diet group * Time (3 month) | 3.2 (-1.4, 7.7) | 2.3 | 0.18 |
| Combined CBT and FODMAP diet group * Time (3 month) | 1.9 (-2.7, 6.4) | 2.3 | 0.43 |
| CBT group * Time (6 month) | 1.7 (-3.5, 6.8) | 2.6 | 0.52 |
| FODMAP diet group * Time (6 month) | -0.5 (-5.8, 4.8) | 2.7 | 0.86 |
| Combined CBT and FODMAP diet group * Time (6 month) | -2.2 (-7.6, 3.2) | 2.8 | 0.42 |
| **Anxiety (ANX) (n = 552)** | | | |
| Intercept (General patient education group) | 9.4 (8.6, 10.1) | 0.4 | <0.001 |
| CBT group | 0.1 (-0.9, 1.1) | 0.5 | 0.91 |
| FODMAP diet group | 0.6 (-0.4, 1.6) | 0.5 | 0.25 |
| Combined CBT and FODMAP diet group | -0.6 (-1.6, 0.4) | 0.5 | 0.27 |
| Time (3 month) | -0.1 (-0.7, 0.5) | 0.3 | 0.77 |
| Time (6 month) | -0.5 (-1.2, 0.2) | 0.4 | 0.20 |
| CBT group * Time (3 month) | -0.2 (-1.0, 0.7) | 0.4 | 0.68 |
| FODMAP diet group * Time (3 month) | -0.5 (-1.4, 0.4) | 0.4 | 0.28 |
| Combined CBT and FODMAP diet group * Time (3 month) | -0.7 (-1.6, 0.2) | 0.4 | 0.11 |
| CBT group * Time (6 month) | -0.3 (-1.3, 0.7) | 0.5 | 0.57 |
| FODMAP diet group * Time (6 month) | -0.6 (-1.7, 0.4) | 0.5 | 0.21 |
| Combined CBT and FODMAP diet group * Time (6 month) | 0.3 (-0.8, 1.3) | 0.5 | 0.61 |
| **Depression (DEP) (n = 552)** | | | |
| Intercept (General patient education group) | 5.6 (5.0, 6.2) | 0.3 | <0.001 |
| CBT group | 0.0 (-0.8, 0.8) | 0.4 | 0.97 |
| FODMAP diet group | 0.3 (-0.5, 1.1) | 0.4 | 0.46 |
| Combined CBT and FODMAP diet group | -0.7 (-1.5, 0.2) | 0.4 | 0.12 |
| Time (3 month) | 0.2 (-0.4, 0.7) | 0.3 | 0.58 |
| Time (6 month) | 0.3 (-0.3, 0.9) | 0.3 | 0.33 |
| CBT group * Time (3 month) | -0.6 (-1.4, 0.2) | 0.4 | 0.13 |
| FODMAP diet group * Time (3 month) | -0.6 (-1.4, 0.1) | 0.4 | 0.11 |
| Combined CBT and FODMAP diet group * Time (3 month) | -0.4 (-1.2, 0.4) | 0.4 | 0.29 |
| **CBT group * Time (6 month)** | **-1.2 (-2.1, -0.4)** | **0.4** | **0.01** |
| FODMAP diet group * Time (6 month) | -0.8 (-1.7, 0.1) | 0.5 | 0.08 |
| Combined CBT and FODMAP diet group * Time (6 month) | -0.3 (-1.2, 0.6) | 0.5 | 0.52 |
| **Total HADS Score (n = 552)** | | | |
| Intercept (General patient education group) | 15.0 (13.8, 16.1) | 0.6 | <0.001 |
| CBT group | 0.1 (-1.5, 1.7) | 0.8 | 0.93 |
| FODMAP diet group | 0.9 (-0.7, 2.5) | 0.8 | 0.28 |
| Combined CBT and FODMAP diet group | -1.2 (-2.9, 0.4) | 0.8 | 0.14 |
| Time (3 month) | 0.1 (-0.9, 1.1) | 0.5 | 0.88 |
| Time (6 month) | -0.1 (-1.3, 1.0) | 0.6 | 0.81 |
| CBT group * Time (3 month) | -0.8 (-2.2, 0.6) | 0.7 | 0.27 |
| FODMAP diet group * Time (3 month) | -1.1 (-2.5, 0.3) | 0.7 | 0.12 |
| Combined CBT and FODMAP diet group * Time (3 month) | -1.1 (-2.5, 0.3) | 0.7 | 0.11 |
| CBT group * Time (6 month) | -1.5 (-3.1, 0.1) | 0.8 | 0.06 |
| FODMAP diet group * Time (6 month) | -1.4 (-3.1, 0.2) | 0.8 | 0.08 |
| Combined CBT and FODMAP diet group * Time (6 month) | -0.0 (-1.7, 1.6) | 0.9 | 0.96 |
|  | | | |
|  | | | |
