# Supplementary material for: Internet-delivered cognitive behavioral therapy and FODMAP diet for adults with irritable bowel syndrome: A four-arm randomized controlled trial
Source: Internet Interv. 2026 Apr 26;44:100949. doi: 10.1016/j.invent.2026.100949 (PMC13141039; doi:10.1016/j.invent.2026.100949)
Supplement: Supplementary file 8 — CSQ-8 results [file mmc8.docx]

Supplementary file 8 Client Satisfaction (CSQ-8) by Group

|  | | | | | |
| --- | --- | --- | --- | --- | --- |
| ANOVA with partial eta-squared effect size | | | | | |
| Group | N | Mean CSQ-8 (SD) | ANOVA p-value | Partial η |  |
| General patient education | 86 | 22.7 (4.1) |  |  |  |
| CBT | 91 | 23.2 (4.0) |  |  |  |
| FODMAP diet | 90 | 22.6 (4.2) |  |  |  |
| Combined CBT and FODMAP diet | 92 | 23.1 (4.3) |  |  |  |
| Overall Statistics |  |  | p = 0.677 | 0.004 |  |
|  | | | | | |
|  | | | | | |

Note: CSQ-8 scores range from 8-32, higher scores indicate greater satisfaction. Partial η² interpretation: <0.01 = negligible, 0.01-0.06 = small, 0.06-0.14 = medium, ≥0.14 = large. CBT = Cognitive behavioral therapy; FODMAP = fermentable oligosaccharides, disaccharides, monosaccharides, and polyols
